# FRAMe: Empirically informed agent-based modeling of flood resilience in the Mekong River Basin

**DOI:** 10.1016/j.mex.2025.103682

**Published:** 2025-10-17

**Authors:** Wenhan Feng, Liang Emlyn Yang, Mei Ai, Siying Chen, Ziyao Wang, Wenhao Wu, Junxu Chen, Yiping Fang, Yun Xu, Matthias Garschagen

**Affiliations:** a Department of Geography, Ludwig Maximilian University of Munich (LMU), Munich 80333, Germany; b School of Earth Sciences, Yunnan University, 650500 Kunming, China; c Department of Environmental Science and Engineering, Fudan University, 200433 Shanghai, China; d Institute of Mountain Hazards and Environment, Chinese Academy of Sciences, 610299 Chengdu, China

**Keywords:** Agent-based modeling, Flood resilience, Community resilience, Climate adaptive behavior, Social network analysis

## Abstract

The FRAMe (Flood Resilience Agent-Based Model) serves as a framework designed to simulate flood resilience dynamics at the community level, focusing on a rural settlement in the Mekong River basin. Integrating empirical data from extensive surveys, Bayesian networks, and hydrological simulations, the framework quantifies resilience as a trade-off between robustness (resistance to damage) and adaptability (capacity for dynamic response). Agents include households, governments, and other institutional actors, linked by social and governance networks that facilitate knowledge transfer, resource distribution, and risk communication. FRAMe incorporates mechanisms for policy interventions and individual and collective decision-making, grounded in Protection Motivation Theory and MoHuB frameworks. The framework utilizes geographic data to achieve a spatially explicit design. Sensitivity analyses across five key parameter groups covering network structure, knowledge dissemination, and policy intensity demonstrate the model’s robustness and allow systematic evaluation of intervention effects. Simulation results show that increasing the intensity of government assistance reduces average recovery time by around 50 % and improves system-level robustness by about 30 %. In contrast, enhancing emergency relief primarily improves adaptability (approximately 10 %) and shortens recovery time, with limited effect on robustness. Higher knowledge dissemination roughly doubles adaptability but also introduces greater variability in robustness. By examining policy scenarios and agent behavior, FRAMe aims to inform adaptive flood management strategies and foster the improvement of community resilience.

• The agent-based model is described using the ODD+*D* protocol.
• A brief model user's guide is provided.
• The model validation and sensitivity analysis processes that have been conducted are explained.

## Graphical abstract

**Figure:**
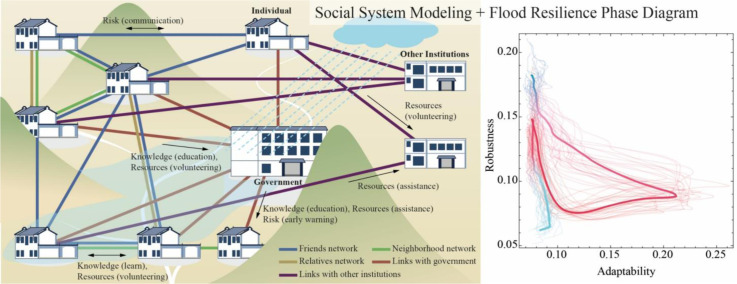

**Specifications table**

**Table:** 

**Subject area**	Environmental Science
**More specific subject area**	*Social simulation*
**Name of your method**	*FRAMe: Flood Resilience Agent-based Model*
**Name and reference of original method**	*NA*
**Resource availability**	*NetLogo is available from:*https://ccl.northwestern.edu/netlogo/, R is available from: https://www.r-project.org, the model code, relevant files, and necessary data can be accessed from: https://www.comses.net/codebase-release/db311523–94f9–4044–8dcc-c208f7707486/

## Background

Resilience, first introduced by ecologist Holling in 1973, has since become a central concept in disaster management, sustainable development, and climate change policy [[1], [2], [3], [4]]. While its definition has evolved over time and interpretations vary across studies, its quantification remains a fundamental challenge [[5], [6], [7]]. Influenced by the widespread use of vulnerability index in the social sciences, indicator-based systems have become the dominant approach [[8], [9], [10]]. This method is clear and intuitive, making it well-suited for regional assessments and cross-sectional comparisons [8,9,11]. However, it also presents notable limitations. On one hand, it struggles to reflect the complexity of social systems; on the other, it fails to capture the dynamic and temporal aspects of resilience. This gap is particularly apparent in governance-related research.

In contrast, agent-based modeling (ABM) offers several distinct advantages [12,13]. It is inherently time-sensitive and process-oriented, allowing for the simulation of dynamic systems over time. ABM can also incorporate social network analysis to represent complex governance structures. By focusing on individual behaviors and interactions, it captures the emergent mechanisms through which resilience arises within social systems [[13], [14], [15]]. Additionally, ABM is well-equipped to handle high levels of uncertainty and enables the integration of empirical data [[16], [17], [18]]. Its flexible modeling platforms further support the simultaneous simulation of environmental changes and social processes [19,20], making it a powerful tool for resilience research.

Previous models have demonstrated the utility of ABMs in capturing diverse behavioral mechanisms and governance dynamics. Some studies have focused on household risk perception and adaptation, emphasizing processes such as risk appraisal, coping appraisal, and social learning (e.g., [15,21,22]). Others have explored behavioral uncertainty and institutional influences, particularly bounded rationality, social norms, and centralized planning, in shaping adaptation outcomes and inequality (e.g., [[23], [24], [25], [26]]).

Compared to existing models, FRAMe (Flood Resilience Agent-Based Model) proposed in this study introduces several distinct contributions. First, while previous studies have advanced the integration of human behavior into flood simulations, quantitative assessment of social resilience through ABM remains limited. FRAMe addresses this gap by building on the social resilience framework developed by Schweitzer et al. [7,27], which conceptualizes resilience as a combination of adaptability and robustness. The model operationalizes this concept and dynamically visualizes system trajectories in a continuous phase space, moving beyond indirect or one-dimensional proxies such as evacuation rates or insurance uptake. Second, FRAMe integrates protection motivation theory (PMT) [28] and the MoHuB framework [29] to simulate how formal interventions (e.g., aid distribution, education programs) interact with individual learning and social feedbacks. Furthermore, it extends prior approaches by integrating social network structures that enable the simulation of knowledge and resource diffusion. Finally, FRAMe couples physical flood processes, using the Manning and Horton equations [30,31], with empirically grounded social behavior through field data and Bayesian network-based synthetic population generation [32]. Applied to a multi-ethnic rural community in the upper Mekong region of Yunnan, China [33], FRAMe also fills a critical empirical and regional gap in a literature still dominated by urban and high-income contexts.

Moreover, while the current application focuses on flood resilience in rural areas of the Mekong River Basin, the modular and data-driven design of the FRAMe model enables its adaptation to other regional contexts. With appropriate empirical data, the model can be extended and calibrated for different geographic settings. The underlying modeling logic and framework of FRAMe can also inform simulations of other risk response behaviors. By customizing or replacing the physical environment module, the framework can be integrated with models of droughts, landslides, or heatwaves, thereby enhancing its relevance to broader climate resilience research.

Broader discussions in the ABM community underscore the importance of structured reporting protocols, replication standards, and modular open-source platforms to enhance transparency and reproducibility [[34], [35], [36]]. In response to these developments, the Method Details section adopts the ODD+*D* protocol [37] to systematically describe the model structure and provides practical guidance on model usage, parameter adjustment, and output analysis. The Method Validation section discusses the minimum number of simulation runs required to address model uncertainty, as well as model calibration and sensitivity analysis. These choices also reflect ongoing debates on fit-for-purpose modeling and the pragmatic integration of theory in ABM, which emphasize balancing analytical rigor, practical usability, and cautious generalization [38,39].

## Method details

### Overview

#### Purpose

FRAMe is developed to model the resilience of human social systems in the context of flood disasters and to test strategies for enhancing it. Based on its use of survey and geospatial data, FRAMe is configured for a mountainous village settlement (referred to as TP Village) in the upper Mekong River Basin. With this model, we can study the combined impact of flood events, local governance, and social networks on community resilience. This supports a more systematic understanding of human community resilience and contributes to the development of behavioral theories and quantitative modeling methods related to flood resilience. The model also enables simulations of different policy intervention scenarios, such as the effects of education, aid, and insurance. In particular, it focuses on the distribution of responsibilities between individuals and governments and the role of social networks. It can also be applied more broadly to engage stakeholders in disaster risk communication and learning.

### Entities, state variables, and scales

#### Entities and state variables

**Fig. 1:**
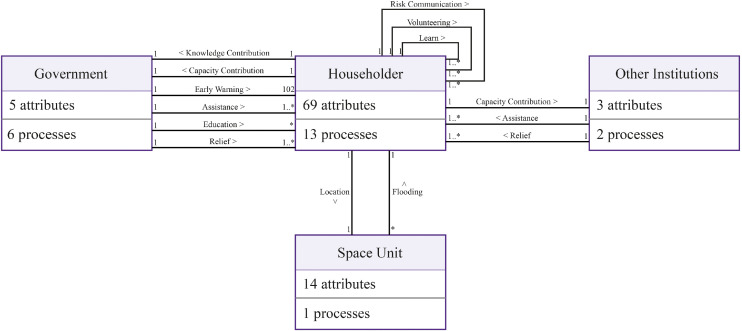
UML class diagram of key entities and their interactions.

The model consists of five types of entities: households (hhs), governments (govs), other institutions (oas), spatial units (Patches), and social links (links). These entities represent the key components of the community flood resilience system. Their interactions simulate how flood impacts, behavioral adaptation, and policy interventions co-evolve during and after flood events. The UML class diagram (Fig. 1) outlines the fundamental characteristics and relationships among these entities [40].

Households are the primary decision-making agents in the model. They take actions at different stages of flooding, including pre-flood protection, in-flood response, and post-disaster volunteering during the recovery period. To reflect the complexity of their roles, the model assigns households a comprehensive set of attributes and behavioral processes. These attributes are categorized into six groups (Table 1): demographic and social characteristics, psychological and knowledge attributes, social capital, behavioral dynamics, risk and resilience indicators, and initialization-related variables. In addition, the model includes several adjustable parameters to support scenario design and model calibration. Each category plays a specific role in shaping household behavior and resilience outcomes. For instance, demographic and social characteristics such as income level or household composition influence individual cognition and value orientation. Psychological and knowledge attributes such as risk perception and self-efficacy determine the likelihood and scope of protective actions a household may adopt. Higher levels of perceived risk and self-efficacy are associated with an increased probability of taking early protective measures. Social capital attributes describe how households are embedded in social networks, such as the strength of ties with neighbors or local institutions, which affects access to support and the diffusion of adaptive behaviors.

**Table 1:** Attribute variables of household agents.

Category	Name in model	Description	Mathematical form	Value range
Demographic and social characteristics	own	The value of household-owned assets (including property, vehicles, appliances, and other possessions), normalized into five levels	Ordinal Categorical	[1, 5]
	iden	Self-assessed sense of belonging within the community	Ordinal Categorical	[0, 4]
	popu	Number of working-age household members (excluding elderly and children)	Integer	(0, +∞)
	old	Number of elderly members in the household	Integer	(0, +∞)
	kind	Number of children in the household	Integer	(0, +∞)
	unhealth	Presence of people with health issues in the household	Boolean	0 -> No, 1 -> Yes
	edu	Highest level of education attained within the household	Categorical	0 -> No one has ever been to school, 1 -> Primary school, 2 -> Junior high school, 3 -> Senior high school (technical secondary school), 4 -> Undergraduate, 5 -> master degree, 6 -> Doctor degree or above
	race	Ethnicity formed based on different cultural backgrounds	Categorical	0 -> Han, 1 -> Yi, 2 -> Lisu, 3 -> Undetermined ethnicity
	income	Monthly income, categorized into 9 levels	Ordinal Categorical	[1, 9]
Psychological and knowledge attributes	selfe	Self-assessed self-efficacy	Floating-point number	(0, 1)
	riskp	Self-assessed risk perception	Floating-point number	(0, 1)
	quac	Quantified value for the "Resource Capacity" dimension of individual knowledge	Floating-point number	(0, 1)
	qgac	Quantified value for the "Environmental Needs" dimension of individual knowledge	Floating-point number	(0, 1)
	devec	Quantified value for the "Personal Identity" dimension of individual knowledge	Floating-point number	(0, 1)
	collac	Quantified value for the "Adaptive Awareness" dimension of individual knowledge	Floating-point number	(0, 1)
	va	A list of the four quantified value dimensions, representing the individual's overall knowledge profile	List	
	stra-dive1	Diversity of transitional strategies implemented during the flood onset period	Categorical	[1, 8]
	stra-dive2	Diversity of transitional strategies implemented during the flood recession period	Categorical	[1,8]
	stra-dive3	Diversity of building-related technical strategies implemented before the disaster	Categorical	[1, 6]
	stra-dive4	Diversity of livelihood-related technical strategies implemented before the disaster	Categorical	[1, 10]
	stra-dive5	Probability of engaging in voluntary assistance behavior	Categorical	[1, 7]
	know1	Individual's transition strategies list (before warning)	List	
	know2	Individual's transition strategies list (during floods)	List	
	know3	Individual's pre-flood building technology strategies list	List	
	know4	Individual's agricultural technology strategies list	List	
	know5	Individual's volunteer activity strategies list	List	
Social capital attributes	sn-nb	Indicates whether the individual has a neighborhood network	Boolean	0 -> No, 1 -> Yes
	sn-rl	Indicates whether the individual has a relative network	Boolean	0 -> No, 1 -> Yes
	sn-fr	Indicates whether the individual has a friendship network	Boolean	0 -> No, 1 -> Yes
	sn-gv	Indicates whether the individual has contact with the government	Boolean	0 -> No, 1 -> Yes
	sn-oa	Number of other institutions the individual is connected with	Integer	[0, 4]
	insurance	Indicates whether the individual has insurance	Boolean	0 -> No, 1 -> Yes
	compensation?	Used to indicate whether the individual has already received an insurance payout	Boolean	0 -> No, 1 -> Yes
Attributes related to behavioral dynamics	no-worked-links-list-ind	List of the number of effective links the individual possesses at each tick	List	
	Implemented	Indicator of whether the individual has already taken action within a single tick	Boolean	0 -> No, 1 -> Yes
	social-time	Record of the individual's total social interaction time per day, in hours	Integer	(0, social-time-p-day)
	social-time-p-day	Maximum daily limit of social interaction time for the individual, in hours	Integer	(0, 12)
	no-worked-links-list-ind-from-h	List of the number of effective links the individual has with other individuals at each tick	List	
	powersys-pro?	Indicator of whether the “powersys-pro” strategy has been implemented	Boolean	0 -> No, 1 -> Yes
Attributes related to the quantification of risk and resilience	loss-index	Variable used to quantify individual loss, determined by flood depth and duration, the individual's protection capacity, and loss-coefficient-h ; corresponds to recovery time required, measured in hours	Floating-point number	(0, +∞)
	loss-index-p	Maximum potential loss not accounting for protective measures taken by the individual, measured in hours	Floating-point number	(0, +∞)
	adaptability-ind	Individual adaptability	Floating-point number	[0, 1]
	robustness-ind	Individual robustness	Floating-point number	[0, 1]
	resilience-ind	Individual resilience	Floating-point number	[0, 1]
	recover-time	Actual recovery time of the individual, measured in hours	Integer	[0, +∞)
	flood-stage-h	Flood stage experienced by the individual, determined by the flood stages of all spatial units the individual occupies	Categorical	0->All spatial units are before the flood. 1->The number of spatial units with flood rising is the largest, 2->The number of spatial units with flood falling is the largest, 3->There are no spatial units with flood.
	loss-coefficient-h	Adjustment coefficient for calibrating individual loss	Floating-point number	[0, +∞)
	building-pro-e-t	Probability of building protection possessed by the individual	Floating-point number	[0, 1]
	livelihood-pro-e-t	Probability of livelihood protection possessed by the individual	Floating-point number	[0, 1]
	risk	Individual’s overall perceived risk, representing the probability of taking action	Floating-point number	[0, 1]
	risk1	Risk perception obtained through independent observation of flood dynamics	Floating-point number	[0, 1]
	risk2	Risk observed by others and shared through communication (considered if greater than the individual's own observed risk)	Floating-point number	[0, 1]
	risk3	Risk value based on past flood experience (classified using the natural breaks method)	Floating-point number	[0, 1]
	w-level-m-l	List recording the mean flood depth over the individual’s assets at each tick, assuming no protective measures are taken	List	
Other attributes used for initialization	id	ID number	Integer	[0, +∞]
	assignment	Indicator of whether the household has been assigned attributes	Boolean	0 -> No, 1 -> Yes
	caliduration	Maximum flood duration without considering household action, used for the first layer of the Bayesian network	Integer	[0, +∞)
	calidepth	Maximum inundation depth without considering household action, used for the first layer of the Bayesian network	Floating-point number	[0, +∞)
	caliloss	Actual recovery time of the household, used to calculate loss-coefficient-h, in hours	Integer	[0, +∞)
	r-result	Storage for household attributes estimated by the Bayesian network	List	
	distance-from-other-hhs	List of network distances from this node to other households, used for generating small-world networks	List	
	no-hhfrs	IDs of households with social networks, used for generating small-world networks	Integer	
Adjustable parameters	frequency-learn	Frequency of mutual learning between households, measured in days	Integer	[0, 14]
	responsibility-hh	Household self-responsibility coefficient, representing sense of responsibility	Floating-point number	[0, 1]

Government agents represent local authorities responsible for implementing early warning, assistance, and educational interventions. Their parameters are grouped into fixed attributes (e.g., knowledge libraries) and adjustable policy settings (e.g., aid frequency, targeting mode) (Table 2), reflecting both institutional memory and operational flexibility. These settings capture the top-down dynamics of government behavior, including the design of targeting strategies, aid distribution, and engagement frequency with households. Governments connect with households through networks and can provide them with resources and support.

**Table 2:** Attribute variables of government agents.

Category	Name in model	Description	Mathematical form	Value range
Attribute	best-build-s	ID of the optimal building-related strategy	Categorical	[1, 6]
	best-live-s	ID of the optimal livelihood-related strategy	Categorical	[1, 10]
	contribution-library-b	Household contribution to the government in the area of building protection, which can be transferred to benefit other households	Floating-point number	[0, +∞)
	contribution-library-l	Household contribution to the government in the area of livelihood protection, which can be transferred to benefit other households	Floating-point number	[0, +∞)
	id	ID number	Integer	[0, +∞)
Adjustable parameters	frequency-education	Frequency of educational programs, measured in days	Integer	[0, 28]
	va-edu?	Whether the educational program includes value-based education	Boolean	0 -> no, 1 -> yes
	edu-duration	Duration of a single educational session, measured in hours	Integer	[1, 12]
	assis-mode	Targeting mode for pre-disaster assistance and emergency relief during and after the disaster	Boolean	“Random-assis” refers to random search, while “target-assis” refers to targeted search for more vulnerable households.
	frequency-assis-local	Assistance frequency of the local government, measured in days	Integer	[0, 28]
	assis-local-proportion	Assistance coverage ratio of the local government	Floating-point number	[0.1, 1.0]
	frequency-assis-hl	Assistance frequency of the higher-level government, measured in days	Integer	[0, 28]
	assis-local-proportion	Assistance coverage ratio of the higher-level government	Floating-point number	[0.1, 1.0]
	human-no-emergency relief	Maximum number of personnel that can be mobilized in a single emergency relief action during or after the disaster	Integer	[1, 10]
	frequency-emergency relief-local	Emergency relief frequency of the local government, measured in days	Integer	[0, 7]
	emergency relief-local-proportion	Emergency relief coverage ratio of the local government	Floating-point number	[0.1, 1.0]
	frequency-emergency relief-hl	Emergency relief frequency of the higher-level government, measured in days	Integer	[0, 7]
	emergency relief-h-l-proportion	Emergency relief coverage ratio of the higher-level government	Floating-point number	[0.1, 1.0]
	insurance-plan?	Whether a mandatory insurance scheme exists	Boolean	0 -> no, 1 -> yes
	insur-p-proportion	Proportion of the population enrolled in the mandatory insurance scheme	Floating-point number	[0.1, 1.0]
	compensation-ratio	Compensation ratio of insurance payouts relative to losses	Floating-point number	[0.1, 1.0]

Other institutions include local organizations such as clan groups or financial institutions. They act as complementary actors in resilience building, providing additional support or facilitating coordination when government assistance is limited or delayed. Their structure parallels that of government agents (Table 3), enabling comparative simulations of institutional support under varying conditions.

**Table 3:** Attribute variables of other institutions agents.

Category	Name in model	Description	Mathematical form	Value range
Attribute	contribution-library-b	Household contribution to the organization in the area of building protection, which can be transferred to benefit other households	Floating-point number	[0, +∞)
	contribution-library-l	Household contribution to the organization in the area of livelihood protection, which can be transferred to benefit other households	Floating-point number	[0, +∞)
	id	ID number	Integer	[0, +∞)
Adjustable parameters	assis-mode	Targeting mode for pre-disaster assistance and emergency relief during and after the disaster	Boolean	“Random-assis” refers to random search, while “target-assis” refers to targeted search for more vulnerable households.
	frequency-assis-local	Assistance frequency of the organization, measured in days	Integer	[0, 28]
	assis-local-proportion	Assistance coverage ratio of the organization	Floating-point number	[0.1, 1.0]
	human-no-emergency relief	Maximum number of personnel that can be mobilized in a single emergency relief action during or after the disaster by the organization	Integer	[1, 10]
	frequency-emergency relief-local	Emergency relief frequency of the organization, measured in days	Integer	[0, 7]
	emergency relief-local-proportion	Emergency relief coverage ratio of the organization	Floating-point number	[0.1, 1.0]

Spatial units are represented as grid-based patches that encode environmental attributes such as elevation, land use, and water depth (Table 4). These parameters determine the spatial heterogeneity of flood and shape how agents perceive and respond to flooding.

**Table 4:** Attribute variables of spatial units.

Category	Name in model	Description	Mathematical form	Value range
Attribute	is-road?	Land use type	Categorical	0 -> Impervious, NaN -> pervious
	dem1	Digital elevation data used for flood simulation, in meters	Integer	(-∞, +∞)
	is-public?	Used to distinguish between public (government) and private (household-owned) buildings at this location	Categorical	0 -> hh, 1 -> gov
	no-building	ID numbers of household and government buildings	Integer	[0, +∞)
	flood-stage	The flooding stage currently experienced by the patch	Categorical	0 -> pre, 1 -> during flood rise, 2 -> during flood decline, 3 -> post
	Nmanning	Patch's Manning’s friction coefficient	Categorical	Impervious -> 0.3, pervious(crop) -> 0.035
	Infiltration	Indicates the reduction in water volume on the patch due to infiltration and evaporation	Categorical	Impervious -> 0.2, pervious -> 0.5
	w-level	Current water height on the patch, in millimeters	Floating-point number	[0, +∞)
	w-level-last	Maximum historical water height on the patch, in millimeters, used to distinguish between flood rise and recession phases	Floating-point number	[0, +∞)
	w-level-list	Historical list of water heights on the patch, in millimeters	List	
	flood-time	Flood onset time	Integer	[0, +∞)
	wl-infiltration	Infiltrated water volume, in millimeters	Floating-point number	[0, +∞)
	duration-flood	Duration of the flood, in hours	Integer	[0, +∞)
	risk-map	Risk value determined solely by geographic location (hazard-based only)	Floating-point number	[0, +∞)

Social links represent the informal and formal relationships among all types of agents. The model distinguishes between household–household (e.g., neighbors, relatives, friends) and institution–household connections (Table 5). These links form dynamic networks through which knowledge, behaviors, and resources circulate.

**Table 5:** Attribute variables of links.

Category	Name in model	Description	Mathematical form	Value range	Remark
Attribute	worked-time	Used for timekeeping, in hours	Integer	[0,+∞)	
	worked	Indicator of whether the link is active	Boolean	0 -> no, 1 -> yes	
	worked-time-req	Duration of effectiveness, measured in hours	Integer	[0,+∞)	
	rewired?	Used for generating a small-world network	Boolean	0 -> no, 1 -> yes	Only friend links have

#### Exogenous factors / drivers of the model

A flood simulation is incorporated to capture flood formation, propagation, and recession based on terrain, land use, and rainfall patterns. Elevation, defined by a digital elevation model (DEM), determines water flow direction and speed. Land use influence hydrodynamics through friction and infiltration rates. Flood propagation follows Manning’s equation, and infiltration is calculated using the Horton formula . Flood intensity, defined by rainfall intensity and duration, along with the onset timing, can be adjusted within the model.

#### Inclusion of space in the model

Spatial information is embedded in the model in the form of geographic references. The model initializes spatial settings by loading a DEM data, land use data, and building locations. These datasets are used to define the attributes of each spatial unit. A visualization feature is also available to display or hide elevation patterns using color gradients to represent terrain. Geographic references provide realistic context for simulating flood propagation and modeling social dynamics [41].

#### Temporal and spatial resolutions and extents of the model

The model operates with a time step of one hour and simulates a total of 60 days, resulting in 1440 ticks. Each spatial unit measures 12.5 m per side, matching the resolution of the DEM data. The model contains 11,881 spatial units in total (109 cells per side, covering 1362.5 m), representing an area of approximately 1.86 square kilometers.

#### Parameter overview

To improve transparency and replicability, Table 6 presents a consolidated overview of all key parameters used in the model. This includes simulation clocks, global indices, hydrological and spatial constants, and external data inputs related to network generation and risk analysis. Each parameter is listed together with its unit, default value or functional description, and its provenance. Some parameters are used for data storage and for importing information into the simulation environment, such as “data-im” for household attributes and “data-nw” for network structures. Others, like “alist-hh” and “llist-hh”, are designed to track system-level dynamics over time, including changes in adaptability, robustness, and household losses.

**Table 6:** Summary of model parameters.

Category	Parameter Name	Variable	Unit	Default / Description	Source / Justification
Main Program	Simulation hour	hour	hours	Simulation clock (hour)	Used for time tracking
	Simulation day	day	days	Simulation clock (day)	Used for time tracking
	Global adaptability	adaptability	[0, 1]	Ratio of active links	Emergent; network-based
	Global robustness	robustness	[0, 1]	Mean of individual protection probabilities	Emergent; agent-based
	Global loss index	loss-global	[0, 1]	Mean household loss	Emergent output
	Active links record	no-worked-links-list	count	List of active link count over time	For adaptability tracking
	Current active links	no-worked-link	count	# of currently active links	Time-step update
	Resilience index	resilience	[0, 1]	Combined adaptability & robustness	Derived metric
	Flood end time	flood-end	hours	Timestamp of flood end	Computed
	Adaptability from households	adaptability-from-h	[0, 1]	Bottom-up adaptability	Tracked separately
	Adaptability from government	adaptability-from-g	[0, 1]	Top-down adaptability	Tracked separately
	Robustness from households	robustness-from-h	[0, 1]	Bottom-up robustness	Tracked separately
	Robustness from government	robustness-from-g	[0, 1]	Top-down robustness	Tracked separately
	Total compensation	total-compensation	currency units	Insurance payout total	Emergent output
	Adaptability time series	alist-hh	[0, 1]	List of household adaptability	Used for plotting
	Robustness time series	rlist-hh	[0, 1]	List of household robustness	Used for plotting
	Gov. adaptability series	alist-hh-f-g	[0, 1]	From government to households	For decomposition
	Gov. robustness series	rlist-hh-f-g	[0, 1]	From government to households	For decomposition
	Peer adaptability series	alist-hh-f-h	[0, 1]	From peers to households	For decomposition
	Peer robustness series	rlist-hh-f-h	[0, 1]	From peers to households	For decomposition
	Loss index series	llist-hh	[0, 1]	List of household losses	Used for output comparison
	Loss index w/o protection	lplist-hh	[0, 1]	Baseline loss values	Used as benchmark
Basic Environment Module	DEM data	dem	meters	Digital Elevation Model	Spatial input
	Building polygons	building-location	polygon shapefile	Building SHP polygons	Spatial input
	Land use map	road	polygon shapefile	Road/non-road classification	Spatial input for Manning
Hydrological Environment Module	Rainfall intensity	rain-intensiv	mm/hour	Applied to grid	Hydrological input
	Rain duration	t-rain	hours	Rain duration	Scenario parameter
	Grid cell length	r-m	meters	Grid size for Manning	Model config
	Grid cell area	r-m2	m²	Computed from r-m	Model config
Population Synthesis Module	Max resource capacity	mquac	normalized unit	Normalization value	From population
	Max environmental demand	mqgac	normalized unit	Normalization value	From population
	Max identity value	mdevec	normalized unit	Normalization value	Survey-based
	Max adaptation awareness	mcollac	normalized unit	Normalization value	Survey-based
	Knowledge (pre-warning)	know-base1	index [1, 8]	8 strategies	Household input
	Knowledge (during flood)	know-base2	index [1, 8]	8 strategies	Household input
	Knowledge (building tech)	know-base3	index [1, 6]	6 strategies	Household input
	Knowledge (agri. tech)	know-base4	index [1, 10]	10 strategies	Household input
	Knowledge (volunteer)	know-base5	index [1, 7]	7 strategies	Household input
	Household attributes	data-im	CSV file	CSV input	Input file
	Network data	data-nw	CSV file	CSV input	Input file
	Small-world param	Infinity	dimensionless	Network generation constant	Used in Watts-Strogatz model
	Attribute ratios	r1-result	proportion	Proportion matrix	Computed from R
	Calibration data	data-im1	CSV file	CSV calibration coefficients	For model tuning
Population Synthesis Module (Constructing a small-world network)	Lattice path length	average-path-length-of-lattice	steps	Initial network path length	Network metric
	Current path length	average-path-length	steps	Updated path length	Network metric
	Rewired edge count	number-rewired	count	# of rewired edges	Network tracker
	Householder set	hhfrs	agent set	Agents with networks	Agent subset

### Process overview and scheduling

#### Overall process

At each tick of the simulation, households first determine their current flood stage based on the extent to which the spatial units containing their assets are inundated. Household responses and the effectiveness of their actions vary across different flood stages. Perceived risk is the primary driver for strategy implementation [28], while outcomes also depend on individuals’ self-assessed capacity and cognitive factors such as knowledge and values [42,43]. In addition, households may also engage in social learning and volunteering within their networks [44].

**Fig. 2:**
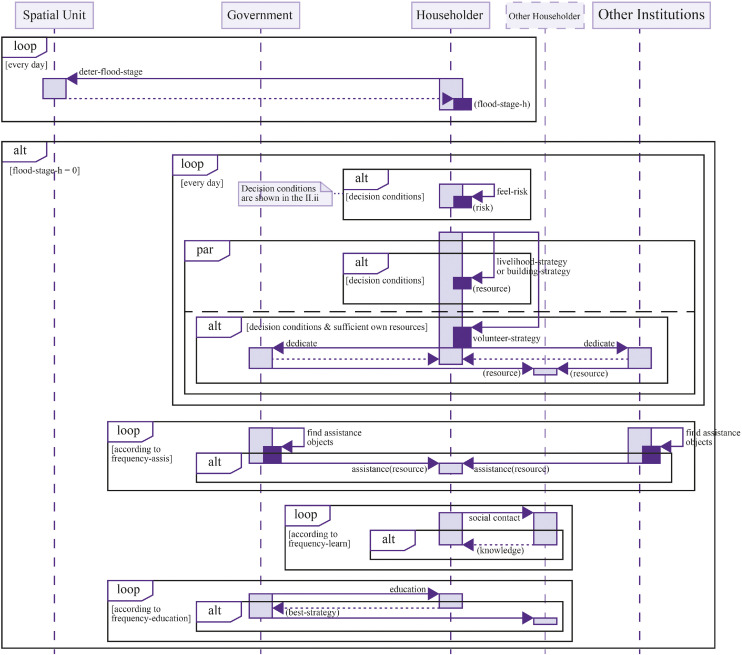
Dynamic response processes before the flood phase.

Governments intervene regularly through education, early warning, insurance, resource assistance, and emergency relief [45]. Other institutions contribute additional support and emergency relief capacities. As shown in Fig. 2, prior to the flood, households may adopt building, livelihood, or volunteering strategies. They can also learn strategies from connected households. In this stage, governments provide education services, identifying and promoting strategies that are most effective in enhancing household protection. Governments and other institutions can also offer direct support to improve individual self-protection capacity.

**Fig. 3:**
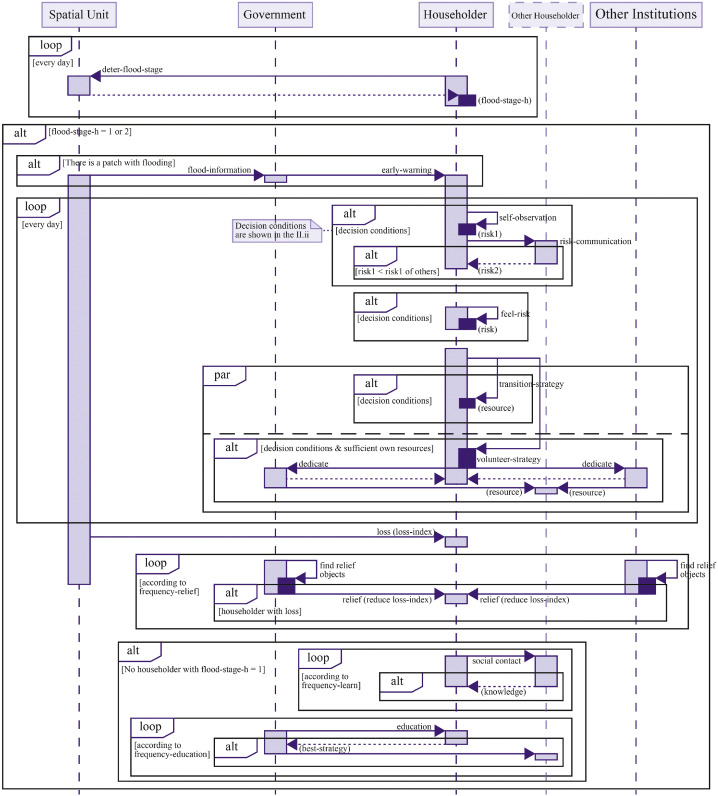
Dynamic response processes during the flood phase.

As shown in Fig. 3, during the flood phase, the model dynamically updates flood conditions such as water level, direction of spread, and speed. Households assess their risk based on their own observations and communication within their social networks, and implement possible transitional strategies. This phase can be further divided into the period of flood onset and the period of flood recession, during which the effectiveness of household strategies varies. When the flood starts to rise, the government immediately provides early warning services. At the same time, household social activities, including education services provided by the government, are suspended. Throughout the flood phase, the government carries out emergency relief operations when needed, offering broader support to the community and accelerating recovery. Capable households also assist others within their social networks. The model includes a “loss-index”, corresponding to the time required for recovery (in hours). The model calibration process is specifically aimed at calibrating this “loss-index”.

**Fig. 4:**
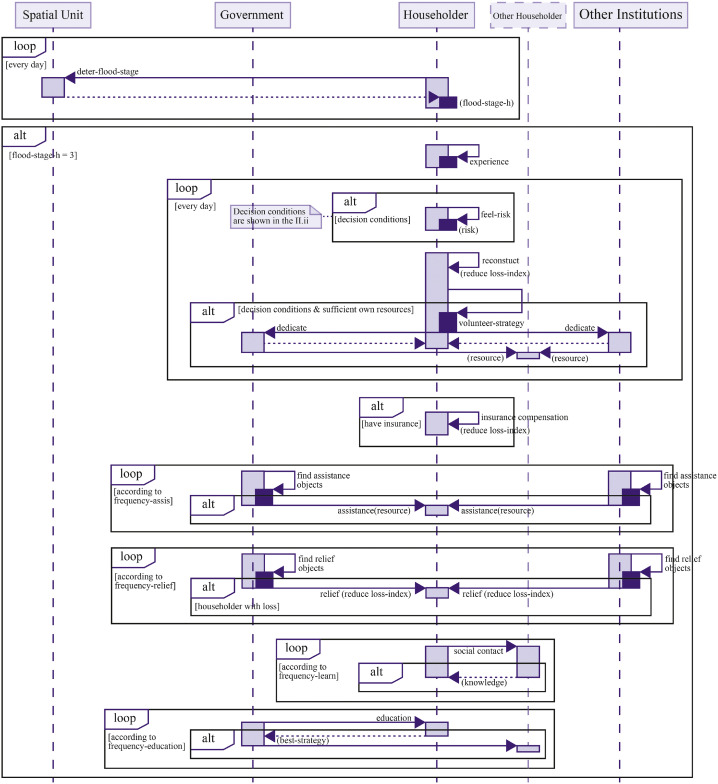
Dynamic response processes after the flood phase.

As shown in Fig. 4, at the end of the flood, insured households receive compensation, which helps shorten their recovery time. After the flood, households adjust their behavioral patterns based on the extent of their losses. Households also actively engage in recovery efforts, assisting either themselves or others within their social networks. As long as there are households that have not yet recovered, the government continues to provide emergency relief services. Once all households have recovered, the model returns to the pre-flood operational steps.

#### Process on social networks

In the model, the role of social networks is explicitly integrated into the interaction processes between households and institutions. The model includes a network of governance links between households and government actors (including other institutions). Households also possess their own horizontal social networks, consisting of friends, neighbors, and relatives. Each type of network plays a specific role during different phases of the flood event, supporting household responses. These processes mainly revolve around knowledge dissemination, resource sharing, and risk information communication.

• Knowledge dissemination process: On one hand, the government identifies effective strategies through its connections with households and spreads these strategies. On the other hand, households can directly acquire new response strategies and enhance their awareness through learning within their friends, neighbors, and relatives.
• Resource sharing process: During volunteer-based activities, households provide support to those in need through their friends, neighbors, and relatives. In aid efforts, the government and other institutions deliver support through their direct links to households.
• Risk information communication process: Risk communication is a key mechanism for dynamically adjusting risk perception. Households share flood risk information through their social networks. And the government provides early warning services.

**Fig. 5:**
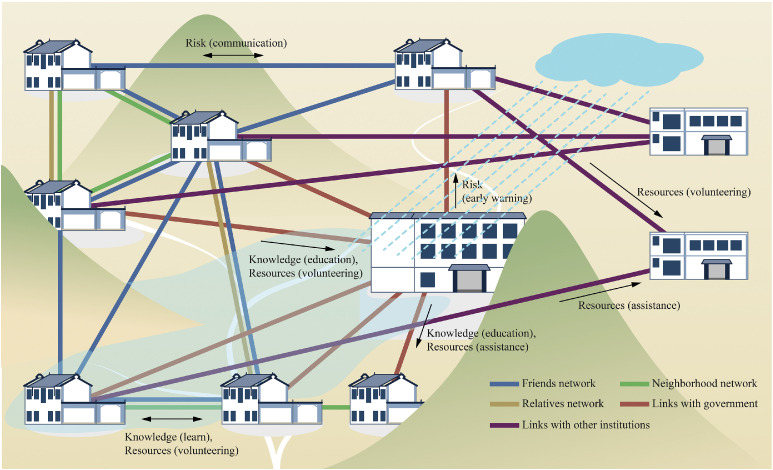
Conceptual representation of social networks and their functions in the model.

Fig. 5 conceptually illustrates the role of social networks in the model, showing household connections with different types of networks and how these connections influence the dissemination of knowledge, resources, and risk information.

## Design concepts

### Theoretical and empirical background

The model is a novel extension of a resilience quantification framework specifically designed for unstable and non-equilibrium social organization [7], applying it to community resilience under flood impacts. This framework is purposefully developed for use in agent-based modeling and integrates social network analysis. In this framework, social resilience is conceptualized as a trade-off between two dimensions: robustness and adaptability. These two indicators are the key measures observed in our model.

### Individual decision making

In the model, households, the government, and other institutions all possess decision-making capabilities. Government and institutional decisions are primarily driven by predefined parameters or environmental conditions and exert direct influence on households. In contrast, household decision-making is more complex, shaped by spatial factors, individual attributes, and social networks.

**Fig. 6:**
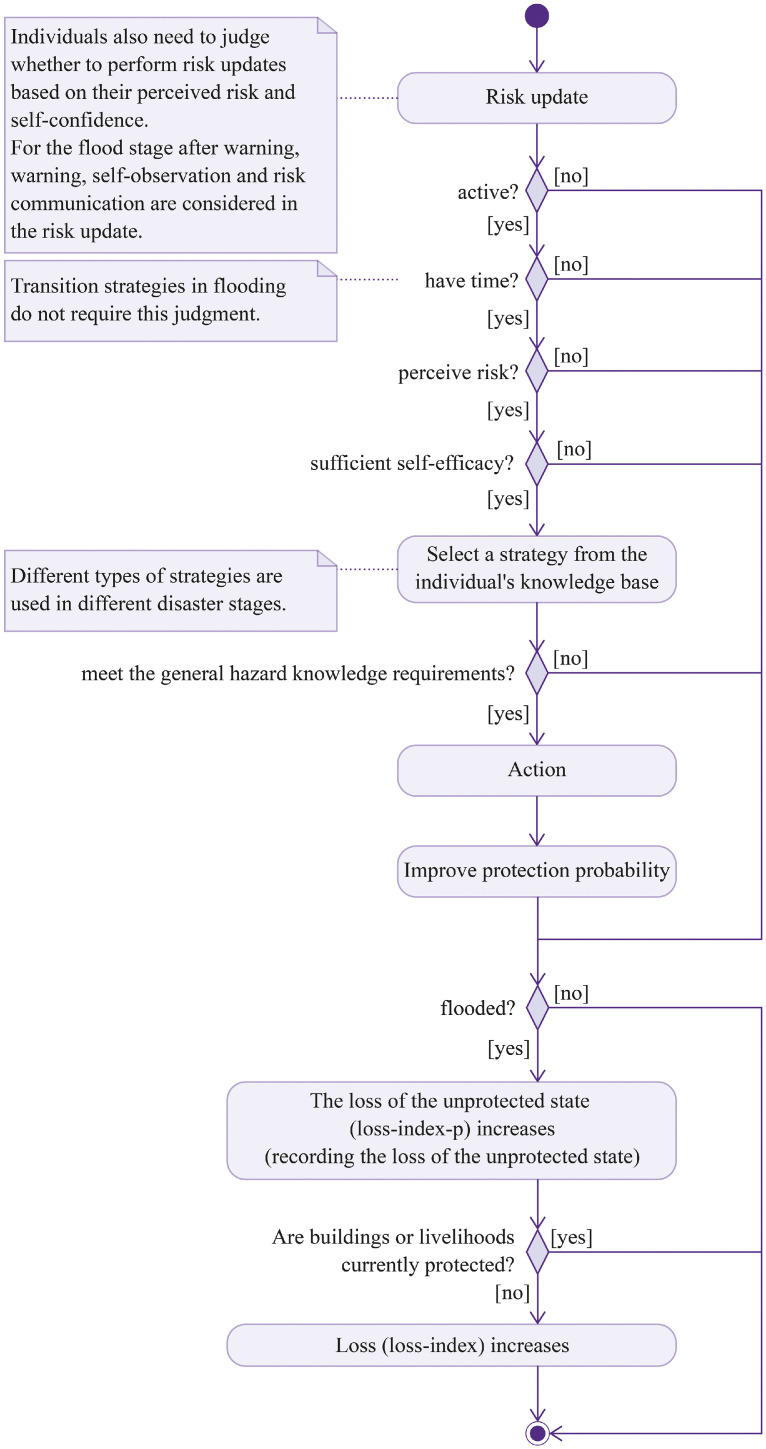
Household decision-making process.

Their decision-making mechanisms draw on the MoHuB framework and PMT, while incorporating the concept of capability–action gaps resulting from cognitive limitations [28,29,43]. These two frameworks are selected because they offer robust explanations of household behavioral dynamics in response to flood risk. PMT, widely applied in disaster preparedness research, provides a structured lens for understanding the motivations behind individual actions [46]. The MoHuB framework adds further depth by accounting for both internal agent characteristics (such as knowledge) and external contextual factors (such as policy interventions and social connections). Incorporating capability–action gaps allows the model to more realistically capture the diversity of household responses, including both self-oriented and community-oriented behaviors. It is also important to distinguish between the IPCC's formal definition of risk and the perceived risk at the individual level within the model. The household decision-making process is illustrated as a decision tree in Fig. 6. At the theoretical level, this integrative use of multiple frameworks reflects a pragmatic approach to enhancing explanatory power while maintaining empirical grounding, consistent with recent debates in the ABM community [39].

Below is a detailed description of the entire decision-making process:

• Risk update: Households first update their perceived risk, which represents the perceived probability of protection failure. In the absence of flooding, perceived risk gradually decreases over time. If the household is in the flood phase, warning messages, personal observation, and risk communication are incorporated into the risk update. Households also possess a degree of memory and adjust their current risk perception based on past flood experiences [47].
• “Active?”: If the household is currently active, proceed to the next check. Households are active from 6:00 a.m. to 6:00 pm., aligning with typical local routines. This condition is only relevant outside the flood phase. During flooding, households remain active at all times.
• “Have time?”: This step is used to check whether the household is currently engaged in an activity.
• “Perceive risk?”: This represents the household’s threat appraisal. The decision continues only if the household perceives a risk, which is evaluated probabilistically based on the perceived risk.
• “Sufficient self-efficacy?”: This represents the household’s coping appraisal. If the household has insufficient self-efficacy, it will not take further action. This step is also probabilistic and is determined by the “selfe” attribute, which is derived from survey data and generated through a Bayesian network.
• Strategy selection: Based on the current flood phase, the household selects a suitable strategy from its knowledge base. The available knowledge is derived from survey data and generated using a Bayesian network.
• Capacity–action gap: This step simulates the gap between the household’s capacity and actual behavior. Each strategy has associated knowledge conditions, determined through literature review and expert input. If the household’s cognitive level meets the conditions of the selected strategy, the action is carried out. Otherwise, the household is unable to implement the strategy. Cognitive level is derived from survey data and evaluated using an indicator system.
• Action: When a household adopts a strategy, it produces certain effects, such as reducing “loss-index” or enhancing protection capacity.
• Loss assessment: If the household’s area is flooded, the unprotected loss (loss-index-p) is assessed first, representing potential losses in the absence of protective measures. If buildings or livelihoods are protected, the household’s actual “loss-index” does not increase.

All household behaviors, including risk updating, are controlled by this decision process. The decision tree integrates household risk perception, self-efficacy, knowledge, and cognitive capacity, providing a structured framework for analyzing behavior under flood scenarios. Time constraints also play an important role in this process.

### Learning

After a flood event, households record and reflect on its impacts, leading to adjustments in their risk awareness and the duration of their social interactions. In addition, households can also learn strategies and knowledge from their friends, neighbors, and relatives.

### Individual sensing

In the decision-making process, households perceive and consider both endogenous variables (such as available time, risk perception, self-efficacy, knowledge, and value orientation) and exogenous variables (such as floodwater level, inundation extent, and information or knowledge provided by the government and neighbors). Perception may involve errors, such as underestimating or overestimating risk. The spatial scale of perception includes the local spatial unit (defined by GIS data such as the footprint of built assets and line of sight), and the network (social connections), depending on the information transmission mechanism. The mechanisms by which agents acquire information are explicitly modeled, such as through social network communication or by observing the behavior of neighbors.

### Individual prediction

The risk perceived by an agent represents its prediction of whether its current capabilities are sufficient to protect itself in future flood events.

### Interaction

Interactions between agents include both direct interactions, such as services provided by the government, risk communication, and social engagement, and indirect interactions, such as household-to-household socialization occurring continuously within networks or coordination through the government during volunteer activities. Interactions among households, the government, and other institutions take place within social networks, while interactions between spatial units and households occur in geographic space. Communication within these interactions is represented explicitly through information exchange, such as the dissemination of risk messages or the sharing of educational strategies. Different types of networks have distinct structures, which affect how households interact and the strength of those interactions.

### Collectives

Households belong to communities represented by social networks, which are determined by specific attributes possessed by each household.

### Heterogeneity

Households are heterogeneous, with varying attributes across different agents. These attributes are generated from survey data using a Bayesian network combined with spatial location. This heterogeneity leads to differences in behavior, which are reflected in the effectiveness of actions, such as the increased probability of protecting buildings or livelihoods.

### Stochasticity

Most processes in the model are governed by probabilistic mechanisms. First, the Bayesian network method used for population synthesis involves stochastic processes [48].And in each decision round, a household selects a strategy randomly from its available knowledge. The network generation process also involves stochastic elements. For example, rewiring in the creation of small-world networks, the order of link formation in relative network, and the selection of institutional connections by households all involve randomness. When implementing volunteer strategies, the amount of resources provided by a household is randomly determined within its resource capacity. Additionally, the model provides the option for government and other institutional support or emergency relief services to be either randomly assigned or directed toward more vulnerable individuals.

### Observation

Based on the social resilience quantification framework used in our model, the primary simulation outputs collected are global measures of robustness and adaptability. These characteristics are recorded at each tick. Additionally, we focus on the number of days required for recovery, as it is widely recognized in the literature as relevant to resilience [49].

## Details

### Implementation details

The entire model is organized within a single folder. It includes the NetLogo executable file, several data files, and three R scripts. During the simulation process, the model generates two additional files: "newdata.csv" and "coefficient.csv" (sample data for both are included with the model files). The file "newdata.csv" stores the synthesized population data generated using the original data and the Bayesian network. The file "coefficient.csv" contains the calibrated individual-level loss calibration coefficient (“loss-coefficient-h”). The model was developed between May and December 2024.

The main model was developed in NetLogo 6.4 and includes a visual interface panel [50]. The panel contains adjustable parameters that allow for scenario design. The model uses NetLogo’s "SimpleR" extension to communicate with R [51], enabling the implementation of Bayesian networks and natural breaks classification. To run the model, users must first configure the simulation environment. The simulation requires R version 4.4.1 or higher, with the R packages bnlearn [48] (for generating Bayesian networks), gRain [52] (for calculating conditional probabilities), ape and BAMMtools [53] (for computing natural breaks). In some cases, the Rjson package is also required to ensure communication between NetLogo and R [54]. Additionally, users must set the R path in NetLogo according to the requirements of the "SimpleR" extension, as described in the extension documentation [51].

The model is structured using NetLogo’s "__includes" function to ensure clear modularity [55]. Therefore, in addition to the main ".nlogo" file, the model also includes eight ".nls" files. The relative paths for these files are already configured within NetLogo, so users only need to maintain their relative positions for the model to run properly.

In addition, the model uses the NetLogo "GIS" extension (for importing geographic data), the "CSV" extension (for processing CSV data), and the "nw" extension (for network analysis) [56].

**Fig. 7:**
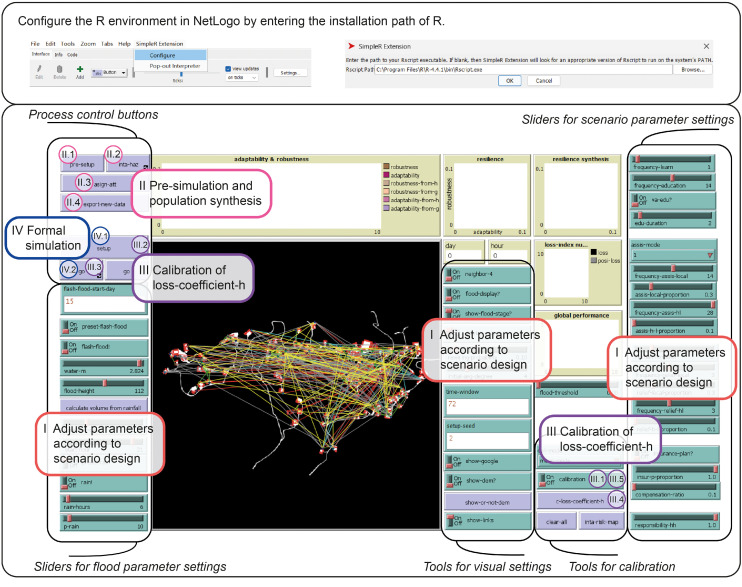
Configuration of R environment and model execution guide in NetLogo.

To run the model, users must first perform pre-simulation and population synthesis steps by sequentially clicking the "pre-setup", "inta-haz", and "asign-att" buttons. Once the synthesis is complete, the "newdata.csv" can be exported to the model folder by clicking "export-new-data". Before the formal simulation, it is necessary to activate the calibration mode and run a complete simulation to calculate the “loss-coefficient-h” (which generates the file "coefficient.csv"). The model can only run properly if the "newdata.csv" exists in the model directory. These procedures may take a considerable amount of time. However, since sample versions of "newdata.csv" and "coefficient.csv" are already included in the model files, users may skip the "pre-setup" steps and directly proceed with "setup". To start the simulation, users should first click the "setup" button to initialize the environment and conditions, followed by the "go" button to run the simulation. Fig. 7 illustrates the complete execution process within the model interface. It includes how to configure the R environment in NetLogo and outlines the sequential steps for running the model: adjusting parameters, generating population attributes, calibrating the loss coefficient, and launching the formal simulation.

### Input data

In our fieldwork across the upper Mekong River Basin, we identified flood-affected villages through prior records and expert consultation. In each village, households were randomly selected for structured interviews, resulting in a total sample of 827 households. The survey instrument collected detailed information on household characteristics, adaptation strategies, social capital, flood experiences, and perceptions. Ethical approval was granted by the Ethics Committee of LMU Munich (Project No 22–0450), and all participants gave informed consent. We obtained five types of data sources:

• Participatory observation, structured household interviews, and survey data: These were used to determine individual attributes and to describe the local flood governance system.
• Expert questionnaires, interviews, and workshops: These supported the estimation of strategy and policy effects within the model, the initial design of the household survey (especially for knowledge and strategies), and the structure of the Bayesian network.
• Geographic information data: This includes the locations of buildings and roads, which were recorded through field observation and mapped in GIS. DEM data was obtained from NASA.
• Locally relevant grey literature: From this, we obtained records related to the 2015 flood event in TP Village, including total rainfall, duration, and the emergency response process.
• Scientific literature: Hydrological parameters used in the flood model were derived from scientific studies.

The household questionnaire covered a broad range of topics beyond those directly used in the model, including livelihood transitions, health, disaster history, and policy preferences. For the purpose of model parameterization and calibration, we extracted 25 key household attributes from specific parts of the questionnaire. Each variable used in the model is explicitly linked to one or more specific survey items, ensuring transparent parameterization and clear behavioral or contextual meaning. This ensures empirical transparency without including the full questionnaire, which can be made available upon request.

Socioeconomic conditions were represented by variables such as “own” (housing and asset value), “income”, “edu”, and “race”, all derived from questions about wealth, monthly income, education level, and ethnic background. Household composition and potential vulnerability were captured through “popu”, “old”, “kind”, and “unhealth”, based on questions about the number of household members in different age groups and the presence of health issues. Social capital and access to informal support were represented by “iden”, derived from four binary questions about community engagement and perceived social ties.

Five variables (“stra-dive” 1–5) captured different categories of household strategies reported by respondents, including post-warning actions, during-flood actions, physical preparedness, livelihood adaptation, and reliance on external help. Each of these was computed by summing binary responses to multiple strategy items. Flood exposure and impact were represented by “caliduration”, “calidepth”, and “caliloss”, based on self-reported water depth, flooding duration, and repair time.

We also included two perception-related variables: “selfe”, derived from six Likert-scale questions reflecting expected losses and planned responses, was designed to capture low self-efficacy and lack of proactive adaptation willingness; and “riskp”, capturing perceived flood protection and institutional trust, based on five attitudinal questions.

Five binary or categorical variables (“snnb”, “snrl”, “snfr”, “sngv”, and “snoa”) were constructed from multiple-choice questions asking about the sources of help during and after floods. These represent different forms and diversity of social support networks. Insurance, a binary variable, reflects whether the household purchased flood-related insurance, indicating institutional protection. Each of these variables thus maps directly to survey content and represents theoretically meaningful factors affecting household-level adaptive and recovery behavior.

To facilitate reproducibility and empirical transparency, the processed anonymized data corresponding to the 25 household variables used in the model, along with the full model code and documentation, are publicly available at the CoMSES Net platform: https://www.comses.net/codebase-release/db311523-94f9-4044-8dcc-c208f7707486/

Below, we explain the relevant survey questions and data processing methods.

1. “Own”: Estimated by summing responses to two asset-related questions. Each type of asset was converted into its equivalent value in RMB. The total value was then divided into five equal categories, ranging from 1 to 5.
● *"If you had to completely rebuild your building (house), how much would it cost in RMB? Or, how much could your house be sold for now (excluding land value)?"*
● *"The value of household assets such as appliances and furniture."*
2. “Iden”: Calculated as the sum of four responses to questions related to social capital. These questions follow commonly used formats in social capital research [57]. Each question is scored as either 0 or 1, resulting in a total score ranging from 0 (none of the responses are positive) to 4 (all responses are positive). The final variable therefore has five possible values, from 0 to 4.
● *"In the past 10 years, have you actively participated in local community activities (such as poverty alleviation programs, policy promotion events, or festivals)?" 0: No; 1: Yes*
● *"Are you a manager or organizer of any local groups or organizations?" 0: No; 1: Yes*
● *"If you had to leave for a day, is there someone who could help take care of your house or children?" 0: No; 1: Yes*
● *"Do you feel that you are a part of the community where you live?" 0: No; 1: Yes*
3. “Popu”:
● *"How many people aged 15 to 64 are there in your household?"*
4. “Old”:
● *"How many people aged 65 or older are there in your household?"*
5. “Kind”:
● *"How many people aged 0 to 14 are there in your household?"*
6. “Unhealth”:
● *"Are there any household members with health issues?" 0: No 1: Yes*
7. “Edu”:
● *"What is the highest level of education attained by any member of your household?" 0: No formal education; 1: Primary school; 2: Middle school; 3: High school (or vocational school); 4: Bachelor's degree (or associate degree); 5: Master's degree; 6: Doctorate or higher*
8. “Race”:
● *"What is your household's ethnic group?" 0: Han; 1: Yi; 2: Lisu; 3: Unidentified ethnicity*
9. “Income”:
● *"What is your household’s available monthly income (in RMB)?" 1:* <*500; 2: 500–1000; 3: 1000–2000; 4: 2000–3000; 5: 3000–5000; 6: 5000–8000; 7: 8000–10,000; 8: 10,000–20,000; 9:* >*20,000*
10. “Caliduration”:
● *"Duration of house flooding (hours)"*
11. “Calidepth”:
● *"Maximum depth (meters)"*
12. “Stra-dive 1″: The questionnaire includes eight types of post-warning transitional behaviors. This attribute represents how many of these eight strategies a household possesses, so the possible responses range from 0 to 8.
13. “Stra-dive 2″: It differs from “Stra-dive 1” only in the timing of the strategy implementation, occurring during the flood period as a transitional behavior.
14. “Stra-dive 3″: The questionnaire includes six types of pre-disaster building techniques. This attribute indicates how many of these six strategies a household employs, so the possible responses range from 0 to 6.
15. “Stra-dive 4″: The questionnaire includes ten types of pre-disaster livelihood behaviors. This attribute indicates how many of these ten strategies a household employs, so the possible responses range from 0 to 10.
16. “Stra-dive 5″: The questionnaire includes seven types of support and assistance behaviors. This attribute indicates how many of these seven strategies a household employs, so the possible responses range from 0 to 7.
17. “Selfe”: The responses to the following six questions use a scale from 1 to 5, where 1 means "very unlikely" and 5 means "very likely." This attribute is the average of their answers, resulting in five possible values ranging from 1 to 5.
● *"How likely are you to suffer economic losses?"*
● *"How likely are you to change your livelihood to earn income in a different way?"*
● *"How likely is the transportation and road system in your residential/work area to collapse?"*
● *"How likely is it that your or your family members’ health will be affected?"*
● *"How willing are you to reinforce and repair your house?"*
● *"How likely are you to relocate (move your residence or business)?"*
18. “Riskp”: The responses to the following five questions use a scale from 1 to 5, where 1 means "strongly disagree" and 5 means "strongly agree." This attribute is the average of their answers, resulting in five possible values ranging from 1 to 5.
● *"This city/region provides good flood protection."*
● *"Your house is relatively safe in future floods."*
● *"Flood warnings issued by local government officials are helpful."*
● *"The government pays attention to implementing good and effective flood control measures."*
● *"Overall, you are satisfied with the flood management in your community."*
19. “Snnb, snrl, snfr, sngv, snoa”: For the four attributes “snnb, snrl, snfr, and sngv”, if a respondent provides an answer corresponding to the respective category in any of the following three questions, the attribute is assigned a value of 1; otherwise, it is 0. For the attribute “snoa”, there are four possible categories. The value of “snoa” is determined by the number of categories selected, ranging from 0 (none selected) to 4 (all selected).
● *"Once you are affected by a flood, who do you think is most capable of helping you (even if they may not have actually helped)? (multiple selections allowed)"*
● *"During disaster recovery, from whom did you receive help? (multiple selections allowed)"*
● *"In your community, which social networks are important for broader and long-term recovery efforts (*e.g.*, rebuilding houses)? (multiple selections allowed)"*
20. “Insurance”:
● *"Do you have flood loss insurance for your house/property?" 0: No; 1: Yes*
21. “Caliloss”:
● *"After the flood event, how many days were needed to repair and restore your assets by yourself?"*

### Initialization

The main purpose of model initialization is to synthesize a virtual community that closely resembles the real world. In this virtual community, household attributes must correspond to the flood hazards and losses they are likely to experience in the spatial dimension. Otherwise, the model’s value for fine-scale geospatial representation is compromised. To achieve this, we first conduct a pre-simulation to obtain the spatial distribution of hazards. Then, using a Bayesian network, we establish the relationship between spatial hazards and household attributes. After assigning attributes to households, we generate social networks based on these attributes and assign corresponding knowledge and knowledge capacity. The detailed explanation is as follows:

#### Flood hazard initialization

Based on DEM data and historical flood records, floods are simulated in the study area to estimate the hazard conditions of each spatial unit within the entire village. Initial risk values are then assigned to households according to inundation levels using the natural breaks classification method. The hazard conditions include two values: maximum inundation depth and inundation duration. These two values serve as the fundamental input data for the Bayesian network.

#### Generate household attributes

**Fig. 8:**
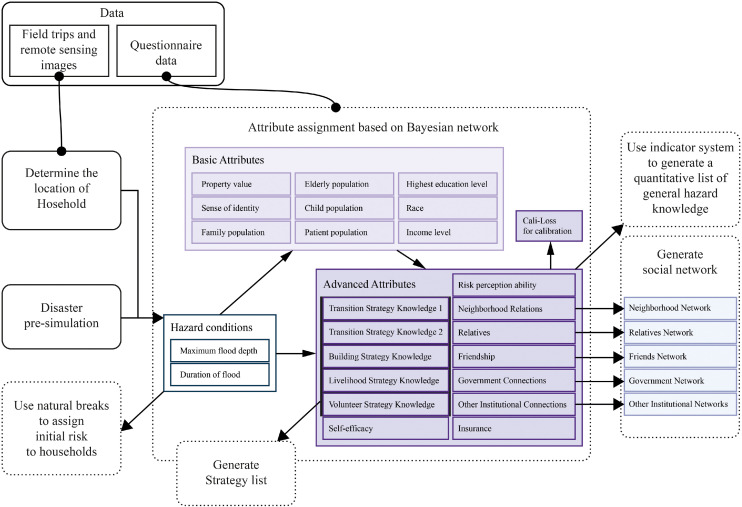
Initialization process diagram.

**Fig. 9:**
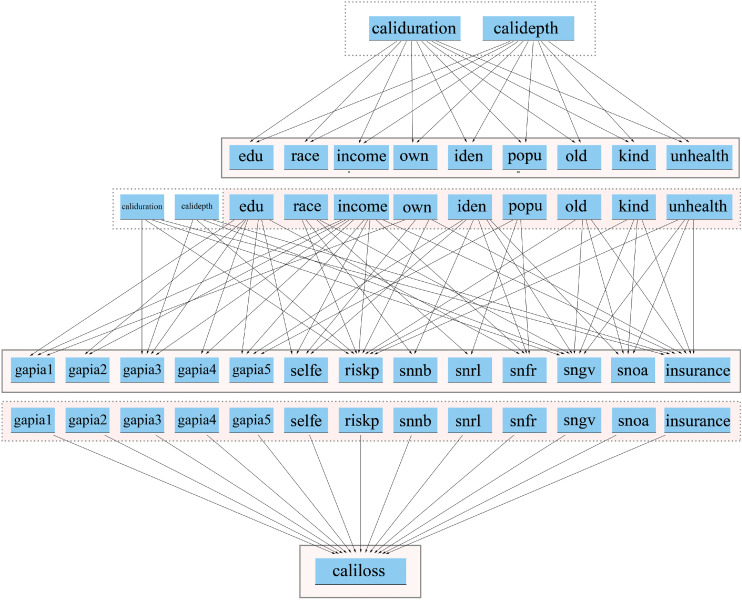
Bayesian network structure.

In the second step, a Bayesian network is used to assign attributes to all household agents. A total of 22 attributes are assigned, divided into two groups (Fig. 8). The first group includes basic demographic characteristics, while the second covers psychological traits, knowledge, and social capital. The Bayesian network consists of three layers. First, it estimates the basic attributes based on hazard conditions obtained from the pre-simulation. Next, it estimates advanced attributes based on both hazard conditions and basic attributes. Finally, advanced attributes are used to estimate an empirically based “loss-index” for calibration (cali-loss). The structure of the Bayesian network was designed through focus group discussions with local experts and stakeholders, a common practice in Bayesian network development [58]. The three networks used are visualized in Fig. 9. In the model, we integrate the R package “bnlearn” for Bayesian computations. “bnlearn” can automatically handle both discrete and continuous attribute types [48].

A Bayesian network is a probabilistic reasoning tool widely used for population synthesis in agent-based modeling [32]. It can flexibly express dependencies among variables under uncertainty and causal relationships [58]. This makes it particularly suitable for modeling complex socio-environmental interaction systems, where household attributes are influenced by external environmental factors (such as hazard conditions) and constrained by endogenous variables (such as risk perception and social capital). This approach provides an interpretable reasoning path for assigning household attributes and ensures that attributes correspond to the flood conditions of their locations. The use of newly generated data preserves respondent privacy while producing household behavioral patterns in the simulation that better reflect real-world empirical data, thus providing a reliable foundation for subsequent decision modeling and analysis [32].

#### Generate social network

The third step involves constructing social networks among households. Each household determines whether it has a certain type of social network based on the social network attributes assigned in the second step. The relative network is determined by the presence of the relative network attribute and ethnic group; every eligible household is linked to other eligible households. The friendship network is determined by the presence of the friendship network attribute and is generated using a small-world network model [59]. The neighborhood network is determined by the presence of the neighborhood network attribute and is based on spatial proximity. This categorical approach to network construction has been used in similar agent-based models [60,61]. The governance network between households and the government is determined by the presence of the government network attribute; all households with this attribute are connected to the government, resulting in a highly centralized network. Similarly, the governance network between households and other institutions involves four types of institutions identified from survey data. Households have an attribute indicating how many other institutions they are connected to, and connections to these institutions are assigned randomly based on that attribute.

#### Assign knowledge, cognitive level and other attributes

Households randomly select knowledge from different knowledge bases based on their respective attributes: stra-dive1, stra-dive2, stra-dive3, stra-dive4, and stra-dive5. Each knowledge strategy has an assigned number, so a household’s knowledge base is stored as a list of these numbers. Cognitive levels are composed of four types of knowledge, which are quantified using an indicator system and normalized to a range of 0 to 1 using the maximum value method [43,62,63]. These four quantified knowledge capacities are also stored as a list. Detailed descriptions of the knowledge and their quantification systems are provided in section III.iv.C.

In addition to knowledge and cognitive levels, the attributes “selfe” and “riskp” are scaled to the range (0,1) using min-max normalization to ensure the model functions smoothly. Another attribute that requires initialization is the daily maximum social time (“social-time-p-day”), which is initially set to 4 based on field observations.

Through the above initialization process, the model establishes a solid foundation for dynamic simulation, ensuring that flood propagation, household decision-making, and social interactions are represented with both realism and complexity. In our case study, at the end of the initialization, the model’s initial state includes 102 households, a government agent, and 4 other institution agents generated based on geographic data. Their state variables are assigned values derived from survey data and the Bayesian network. The attributes of spatial units are determined according to geographic information. Social networks among agents are generated based on their specific state attributes. Due to the involvement of multiple stochastic processes, such as network generation, the initialization is not always identical between runs.

### Submodels

#### Hydrological environment

##### Flood simulation

The flood model is activated after the onset of rainfall (at tick 740 in the model). We use a grid-cell–based flood simulation model primarily designed to simulate flood formation, propagation, and recession [30,64,65]. The model integrates multiple hydrological formulas to dynamically calculate flow paths, flood stages, and the impacts of water levels and infiltration on different land types (such as roads and farmland), allowing for detailed representation of water levels and flood dynamics across spatial units.

First, the model uses Manning’s equation to calculate flow between grid cells:

$$Q_{ij}=\frac{A_{ij}R_{ij}^{2/3}S_{ij}^{1/2}}{n}$$

Where:

• $Q_{ij}$: The flow rate between cells i and j (m³/s)
• *n*: Manning’s roughness coefficient, set according to land type
• *A*: Cross-sectional area
• *R*: Hydraulic radius
• *S*: Water surface slope

The formula used to calculate the flow propagation among various directions is as follows:

$$Q_{flow}=\sum Q_{direction}$$

Next, the model simulates infiltration within each grid cell using Horton’s equation [66]:

$$WL_{infiltration}=f_{c}+\left(f_{o}-f_{c}\right)e^{-\beta t}$$

Where:

• $WL_{infiltration}$: Instantaneous infiltration rate (mm/h)
• $f_{c}$: Final infiltration rate
• $f_{o}$: Initial infiltration rate
• β: Decay constant
• *t*: Time (in hours)

**Fig. 10:**
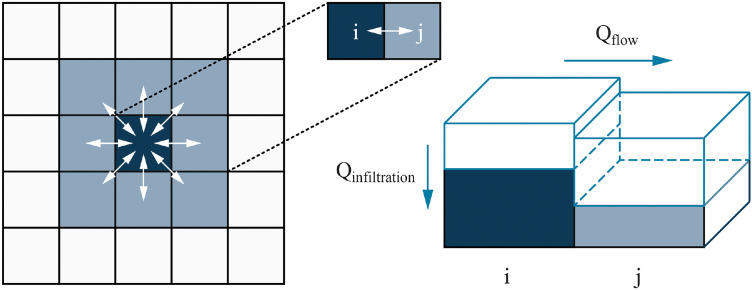
Flood simulation diagram.

At each time step, the instantaneous infiltration rate is calculated using this formula and subtracted from the current water level to update the water depth of the grid cell (Fig. 10).

The product of $WL_{infiltration}$ and the cell area $A_{cell}$ is the amount of water infiltrating $Q_{infiltration}$. Water level update formula is as below:

$$\frac{dWL}{dt}=\frac{Q_{flow}}{A_{cell}}-WL_{infiltration}$$

##### Flood stage determination

The determination of the flood stage is based on the current cell water level (“w_level”), the previous time step water level (“w_level_last”), a flood threshold (“threshold”), and the water levels of neighboring cells. First, if the current water level exceeds the threshold and is rising (higher than the previous water level), the stage is identified as the flood rising stage (Stage 1). If the current water level exceeds the threshold but is falling (lower than the previous water level), it is identified as the flood receding stage (Stage 2). When the current water level is equal to or below the threshold and all neighboring cells also have water levels equal to or below the threshold, if the time is after the flood event, it is classified as the post-flood stage (Stage 3); conversely, if it is before the flood event, it is classified as the pre-flood stage (Stage 0). The flood threshold is generally set to zero, meaning any standing water is considered flooding, but this can be adjusted according to specific contexts.

##### Rainfall design and hydrological parameters

For the rainfall scenario, the model simulates six hours of rainfall using a uniform distribution based on the total precipitation reported in government news for the 2015 flood event. The model also tracks historical water levels through an updated water level list (“w-level-list”) and calculates flood duration for further analysis. Key characteristics of each grid cell are presented in Table 7. Input parameters consist of rainfall intensity and rainfall duration.

**Table 7:** Hydrological parameters.

Land use type	*n*	$f_{c}$	$f_{o}$	β
Paved surface (road)	0.3	25	210	2.0
Unpaved surface	0.035	290	900	0.8

##### “loss-index” calculation

First, the model determines the average water level (“w-level-t”) in the area where the household's property is located, representing the intensity of flood impact experienced by the household. When the water level exceeds a predefined flood threshold (“flood-threshold”), the model assesses whether to update the actual “loss-index” depending on the effectiveness of the household’s protection measures. If the effectiveness of building or livelihood protection (“building-pro-e-t” or “livelihood-pro-e-t”) is insufficient (i.e., a random value exceeds the corresponding threshold), the “loss-index” and the “loss-index-p” is updated; otherwise, only the “loss-index-p” is updated.

#### Risk analysis

##### Information sources

Perceived risk is the key factor driving household decision-making in the model. This sub-model simulates how households perceive risk under flood conditions, incorporating mechanisms such as self-observation (“risk1”), risk communication (“risk2”), experience accumulation (“risk3”), and risk evaluation. The main components and processes of the model are described as follows:

• Self-observation: Households perceive risk based on their proximity to floodwaters. The risk value (“risk1”) is calculated using a tiered distance threshold. For instance, if the flood is within 4 units, the risk is assigned a value of 1; if it is >40 units away, the risk drops to 0.4.
• Risk communication: Households share risk information through their social networks, including friends, relatives, and neighbors. If another agent’s “risk1” is higher than their own, a household updates its communicated risk (“risk2”). This process is moderated by each agent’s cognitive traits, namely their values (“quac”), general awareness (“qgac”), and collective concern (“collac”), which determine the probability of sharing perceived risk (“riskp”). The stronger the risk perception, the more frequently communication occurs.
• Experience accumulation: After a flood event, households are classified into different categories based on recovery time, using the Jenks natural breaks method. Each category corresponds to a level of experiential risk (“risk3”), which increases incrementally and influences the timing of subsequent social interactions.

##### Risk update

The risk updating process represents how households in the model integrate risk information from multiple sources and dynamically adjust their perceived risk levels. This process involves the interaction of several factors and reflects how individual risk perception evolves in a changing environment. The formula is as follows:

$$R_{t}\left\{ \begin{array}{c} max\left(R_{1},R_{2},R_{3},R_{t-1}\right)\cdot P_{r}\cdot R_{h}\;if\;max\left(R_{1},R_{2},R_{3},R_{t-1}\right)\cdot P_{r}\cdot R_{h}>R_{t-1}\;\\ R_{t-1}\cdot \delta \;if\;max\left(R_{1},R_{2},R_{3},R_{t-1}\right)\cdot P_{r}\cdot R_{h}\leq R_{t-1}\;and\;flood\_stage\;=\;0\\ R_{t-1}\;if\;max\left(R_{1},R_{2},R_{3},R_{t-1}\right)\cdot P_{r}\cdot R_{h}\leq R_{t-1}\;and\;flood\_stage\;\neq \;0\; \end{array}\right.$$

Where:

• $R_{t}$: Perceived risk at the current time step.
• $R_{1}$: Risk acquired by the individual through self-observation of flood dynamics (“risk1”).
• $R_{2}$: Risk acquired by the individual through communication within their social network (“risk2”).
• $R_{3}$: Risk assessed by the individual based on past flood experiences (“risk3”).
• $R_{t-1}$: Perceived risk from the previous time step.
• $P_{r}$: Household's risk perception capability (“riskp”).
• $R_{h}$: Self-responsibility coefficient used to adjust the perceived risk value (“responsibility-hh”).
• δ: Decay factor used to simulate the gradual decline of risk during non-flood periods.

This risk updating formula integrates multiple sources of risk such as observation, communication, and experience. It uses the maximum value among them to ensure that the highest level of risk is taken as the current reference. The perception probability $P_{r}$ is used to simulate potential loss or misinterpretation during the process of risk transmission. Additionally, a self-responsibility coefficient $R_{h}$ can be applied to reduce the frequency of household responses in the analysis.

If the adjusted composite risk exceeds the value from the previous time step, it is updated to the higher level. If there is no flood (flood-stage = 0) and the risk has not increased, the value is multiplied by a decay factor δ to represent a gradual decline. During flood periods, if the risk does not increase, it remains unchanged.

#### Knowledge and strategy

In the model, knowledge of strategies is stored numerically, inspired by evolutionary economics agent-based models such as SKIN [67,68]. All strategies’ knowledge conditions and effects are encoded. Building strategies and livelihood strategies respectively increase a household’s probability of building or livelihood protection. The “s-community” strategy acts on both friend and neighborhood networks, while the “s-government”, “donate”, and “religion activities” do not directly enhance the capabilities of other households in the household’s personal social network. Instead, they contribute to the capability pools of government and other institutions, which redistribute support through aid actions. Additionally, “s-relatives”, “s-neighbors”, and “s-friends” can reduce the recovery time of assisted households if they have suffered losses. The utility and possible durations of strategies are derived from the average results of three expert focus group discussions (Table 8).

**Table 8:** Strategies and their effects.

Category	Code in Model	Strategy	Description	Strategy Code in Model	Livelihood Protection Effect	Building Protection Effect	Knowledge Requirement	Max Duration (days)
Building technology strategies	Know3	ground-raise	Elevate the foundation.	1	0.03	0.09	1, 3	
		wall	Reinforce walls.	2	0.02	0.05	1, 3	
		door&window	Reinforce doors and windows.	3	0.016	0.036	1, 3	
		roof	Reinforce the roof.	4	0.02	0.033	1, 3	
		structure	Strengthen the overall structure.	5	0.02	0.06	1, 3	
		corrosion-pro	Apply anti-corrosion measures.	6	0.02	0.036	0, 3	
Livelihood strategies	Know4	storehouse	Build a storage facility.	1	0.046	0.016	0, 3	
		diversity	Diversify sources of livelihood.	2	0.076	0.016	0, 3	
		subsidy	Apply for subsidies.	3	0.06	0.02	0, 3	
		seed-tech	Use improved seed technologies.	4	0.04	0.013	0, 2, 3	
		crop-type	Adjust crop types.	5	0.036	0.013	0, 2, 3	
		farm-location	Relocate farmland.	6	0.043	0.016	0, 2, 3	
		mechanize	Adopt agricultural mechanization.	7	0.026	0.013	0, 2, 3	
		dyke	Construct dykes.	8	0.05	0.033	1, 3	
		reclamation	Reclaim land.	9	0.026	0.01	0, 3	
		reseed	Replant crops.	10	0.04	0.01	0, 2, 3	
Volunteer activities	Know5	s-relatives	Support relatives.	1	0.043	0.056	1, 3	12
		s-neighbors	Support neighbors.	2	0.036	0.05	1, 3	8
		s-friends	Support friends.	3	0.033	0.033	0, 3	7
		s-community	Support the community.	4	0.033	0.046	0, 3	8
		s-government	Support the government.	5	0.033	0.04	0, 3	7
		donate	Donate goods or money.	6	0.023	0.04	0, 3	6
		religion	Participate in religious donations.	7	0.023	0.04	0, 3	31
Transition activities 1	Know1	docs-move	Relocate important documents.	1	0.013	0.023	0, 2	
		car-move	Relocate vehicles.	2	0.013	0.05	1	
		furniture-move	Relocate furniture.	3	0.006	0.023	1, 3	
		vpeople-pro	Protect vulnerable individuals (such as children or the elderly).	4	0.02	0.076	1	
		powersys-pro	Shut off the power supply. This action is only performed once during the simulation.	5	0.01	0.05	1, 3	
		sandbag	Use sandbags for blocking.	6	0.03	0.063	2	
		seal	Seal the building.	7	0.02	0.04	1	
		evacuation	Emergency evacuation.	8	0.023	0.073	3	
Transition activities 2	Know2	docs-move	Relocate important documents.	1	0.016	0.036	0	
		car-move	Relocate vehicles.	2	0.01	0.033	1	
		furniture-move	Relocate furniture.	3	0.006	0.023	1	
		vpeople-pro	Protect vulnerable individuals (such as children or the elderly).	4	0.026	0.09	1	
		powersys-pro	Shut off the power supply. This action is only performed once during the simulation.	5	0.001	0.036	3	
		sandbag	Use sandbags for blocking.	6	0.023	0.053	3	
		seal	Seal the building.	7	0.016	0.04	1	
		evacuation	Emergency evacuation.	8	0.033	0.066	3	

Knowledge capacity and cognitive levels are quantified through an indicator system. There are four quantification dimensions derived from expert discussions and literature [62,69]. “Resource Capability (V0)” reflects how individuals or households perceive and prioritize the acquisition and management of resources. “Environmental Needs (V1)” represents the lifestyle and environmental values of household members. “Personal Identity (V2)” captures household members’ recognition of the spiritual and cultural values of their community. “Adaptation Awareness (V3)” embodies the action-oriented value toward natural risks, emphasizing a proactive attitude toward environmental changes.

We constructed the indicator system using household attributes as influencing factors. The selected influencing factors for each dimension are shown in Table 9. Each dimension is aggregated by summation and normalized to a range from 0 to 1 using the maximum value method to convert into conditional probabilities of knowledge capacity. The four dimensions are treated as parallel and stored together in a list.

**Table 9:** Knowledge capability indicator system.

	Dimension of Knowledge	Code in Model	Household Attribute (Influencing Factor)
Knowledge Capacity and Cognitive Levels (C)	V0 Resource Capacity	mquac	own
			edu
			income
			stra-dive4
			insurance
	V1 Environmental Needs	mqgac	popu
			old
			kind
			unhealth
			stra-dive3
			sn-nb
			sn-rl
			sn-fr
			sn-gv
			sn-oa
	V3 Personal Identity	mdevec	iden
			selfe
			riskp
	V4 Adaptive Awareness	mcollac	stra-dive1
			stra-dive2
			stra-dive5

#### Household strategy implementation behavior

**Fig. 11:**
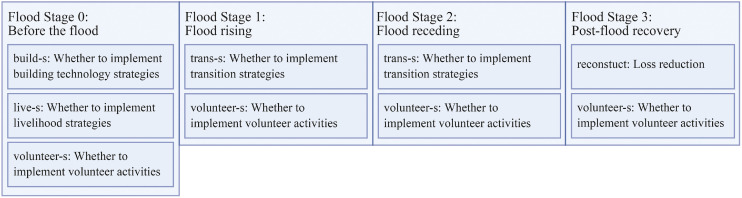
Strategies executable by households at each flood stage.

In the flood model, households adopt a series of strategic behaviors at different flood stages. These behaviors are jointly driven by “risk” and “selfe” and dynamically adjusted as the flood stages progress . The behaviors executable at each stage are shown in Fig. 11.

The entire process of selecting and implementing strategies is divided into three steps. The first step is to identify possible behaviors based on the current flood stage. The second step involves the household’s decision-making process to determine whether to select a strategy for implementation. The third step randomly selects a strategy from the knowledge base list and checks the cognitive conditions; if these conditions are met, the strategy is implemented. Except for the reconstruct behavior, all other behaviors are defined within the knowledge base module. Since the “loss-index” is defined as the time required for recovery, the effect of the reconstruct behavior is to reduce the “loss-index” by one at each tick (hour).

#### Household reflection

Reflection behavior in the model simulates households’ experiential learning and self-adjustment based on past disaster recovery times. This process applies the Jenks natural breaks method from the BAMMtools library [53] to classify recovery times into multiple categories, thereby quantifying the layered influence of experience on risk perception.

First, the model categorizes households’ recovery times using the Jenks natural breaks classification. Based on the assigned category, the household’s experiential risk perception value (“risk3”) is updated. Generally, longer recovery times correspond to higher levels of risk perception [47]. Additionally, households with longer recovery periods tend to spend more time on social interaction and learning after the flood, which is reflected in increased values of “social-time-p-day”. The values of “risk3” and the increments in “social-time-p-day” are detailed in the Table 10:

**Table 10:** Risk perception and social time by recovery time.

Jenks natural breaks category	risk3	social-time-p-day
1	0.25	+0
2	0.4	+1
3	0.55	+2
4	0.7	+3
5	0.85	+4
6	1	+5

#### Household insurance claim

Insurance compensation in the model simulates the risk-sharing and economic reimbursement mechanisms under disaster scenarios. Its core function is to reduce households’ economic losses through insurance payouts. When a household has insurance (“insurance = 1″), insurance proportionally reduces the economic loss caused by the disaster. This reflects the real-world role of insurance in aiding household recovery after a disaster. After the compensation is executed, the variable “compensation?” is set to 1, indicating that the household has received insurance compensation. This prevents duplicate payments and is used to track insurance usage within the model.

#### Household social interaction behavior

Household social behavior is a key mechanism in the model for simulating knowledge dissemination and sharing. At each time step, households interact with others through their social networks, to learn and expand their knowledge. Each household randomly selects a connection from its social network, whether a friend, neighbor, or relative, and attempts to acquire a piece of new knowledge from that connection. If the household does not already possess this knowledge, it adds it to its knowledge base.

#### Assistance and emergency relief actions

Assistance has three sources: local government, higher-level government, and other institutions. In local assistance, assistance is provided based on the availability of resources in the local resource pools (“contribution library b” and “contribution library l”). These resource pools are replenished through households’ volunteer actions. The government can only implement assistance when resources exist in these pools. This design simulates how government aid relies on individual participation when local resources are limited. Assistance increases the target household’s building or livelihood protection capacity. Other institutions provide assistance similarly but with smaller resource allocations. Unlike local assistance, higher-level assistance resources come from external sources, so no resource pools are designed and there are no pool-related constraints. The occurrence of assistance actions depends on assistance frequency parameters (“frequency assis local” and “frequency assis hl”).

The effects of various types of assistance were determined through three expert focus group discussions and are summarized in Table 11:

**Table 11:** Effects of different types of assistance.

Assistance provider	Increase in probability of building protection	Increase in probability of livelihood protection
Local government	0.063	0.06
Other institutions	0.036	0.03
Higher-level government	0.13	0.36

Emergency relief and assistance behavior share similarities and significant differences in their objectives and mechanisms. However, assistance behavior aims to enhance households’ protection capacity, while emergency relief behavior focuses on immediate post-disaster loss mitigation by directly reducing the recovery time to help households recover.

Emergency relief and assistance recipient selection can be random or based on priority rules, such as targeting households with the lowest building or livelihood protection capacity.

#### Educational activities

Government education activities aim to enhance community flood resilience, specifically building and livelihood protection, by sharing optimal strategies and knowledge. Education consists of strategy-oriented training (strategic knowledge) and general education on disaster and environmental knowledge (knowledge capacity and cognitive level). That is to say, this process is not only about knowledge transfer but also optimizes households’ actions by adjusting cognitive level and knowledge capacity.

During the education activity, the government agent first evaluates the flood protection performance of neighboring households and selects those with the best building protection or livelihood protection as learning targets. This includes extracting the best building or livelihood protection strategies from the target households and recording these strategies along with their associated knowledge capacity. The government then shares the learned strategies with other household agents that do not yet possess them. If the cognitive orientation education function (“va-edu”) is enabled, the government also transmits the optimal knowledge capacity to the target households to help them better implement the related strategies.

The conditions for education activities are similar to those for social behavior. However, unlike social interactions, education activities require households to be active and are limited in frequency by the parameter “edu-duration”, which represents the duration of education activities within a day. Additionally, at least one household in the community must have non-zero building protection (“building-pro-e”) and livelihood protection (“livelihood-pro-e”) levels to ensure the educational content is based on existing successful strategies.

#### Mandatory insurance plan

The model also includes a mandatory insurance plan feature, simulated through the insurance plan to represent policy interventions for providing insurance coverage to households. This function selects a portion of uninsured households (“insurance” = 0) to be mandatorily covered based on the insurance coverage rate parameter “insur-p-proportion”, setting their “insurance” to 1.

#### Output parameter calculation and update

The calculation of adaptability reflects the capacity of households to coordinate resources and take action through their social networks. At the household level, each household records the proportion of effective links among all its connections. These proportions are averaged over a defined time window. At the global level, the average of all households’ adaptability indicators is calculated to represent overall adaptability.

The calculation of robustness reflects the system's ability to maintain its original state when facing floods. At the household level, robustness is measured by the average of each household’s building protection efficiency and livelihood protection efficiency. At the global level, overall system robustness is derived by aggregating household data.

The resilience combines robustness and adaptability, using their average to represent the system’s overall capacity. A more effective way to visualize resilience changes is to plot it on a two-dimensional plane with adaptability on the horizontal axis and robustness on the vertical axis. This visualization method serves as one of the model’s primary outputs.

**Fig. 12:**
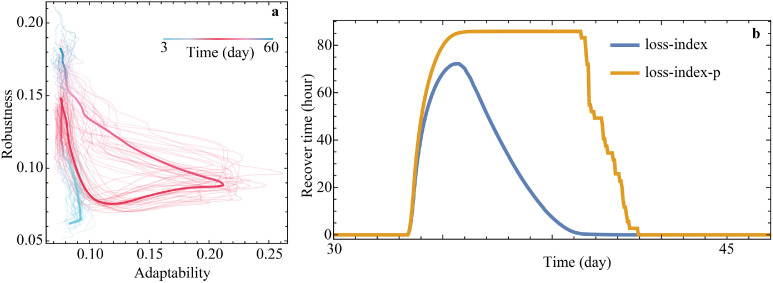
Possible output formats.

As shown in Fig. 12a, each curve represents a dynamic trajectory from one simulation run, with a color gradient indicating time progression. The thick line shows the average trend, while the thin lines correspond to results from thirty individual simulations. During the high-pressure period of the flood scenario, adaptability increases while robustness decreases. This occurs because households rely more on dynamic behaviors, such as social networking and resource coordination, to cope with the crisis, while protective measures are challenged. Fig. 12b depicts changes over time in the “loss-index” and potential “loss-index”. At the flood’s onset, the potential “loss-index” rises sharply, followed by an increase in the actual “loss-index”, highlighting the challenges faced by the system during high-risk periods. As the flood recedes and emergency relief and recovery measures are implemented, the actual “loss-index” begins to decline. The gap between potential and actual “loss-index” reflects the effectiveness of emergency relief and recovery efforts. Because adaptability is calculated over a three-day window, the third day marks the starting point for result outputs.

## Method validation

### Model validation and calibration

To ensure both the empirical plausibility and contextual credibility of the model, we conducted a two-part validation and calibration process. This approach integrates empirical data comparison with stakeholder feedback, following recent calls in the ABM literature emphasizing the importance of reproducibility and robustness through systematic validation and sensitivity analysis [34,35].

First, for empirical alignment, we focused on linking observed recovery behavior with simulated outcomes. Considering that human behavior is continuous, it is difficult to define an exact initial value for the social system over time. The resilience simulated by the model is a relative measure that describes resilience dynamics during the model run. To simulate households’ varying baseline flood protection capacities prior to the simulation period, we designed a coefficient called “loss-coefficient-h”. This parameter connects empirical observations to simulated recovery processes by comparing the empirically derived recovery time (from survey data and Bayesian network inference) with the simulated recovery duration for each household. The loss coefficient for household *i* is defined as:

$$loss\_coefficient\_h_{i}=\frac{caliloss_{i}}{recovery\_duration_{i}}$$

where $caliloss_{i}$ is the empirically derived expected recovery duration for household i and $recovery\_duration_{i}$ is the recovery time produced by the simulation model for the same household. The calibrated “loss_coefficient_h” is then held constant in formal simulation runs to adjust the real-time “loss index” updates, ensuring alignment between empirical expectations and simulated recovery dynamics. This procedure quantitatively anchors the internal damage-recovery process of the model to the empirical recovery distribution. Beyond calibration, this coefficient also plays a conceptual role in linking behavioral diversity and physical exposure. It captures how individual households’ pre-existing protection capacity, informed by socio-economic conditions and local experience, translates into differential resilience performance during floods. In doing so, it provides a consistent scaling mechanism that allows the model to represent both household-level variability and system-level comparability across different contexts. Such empirical anchoring has been highlighted as an important step for building confidence in environmental and social system models [38].

**Fig. 13:**
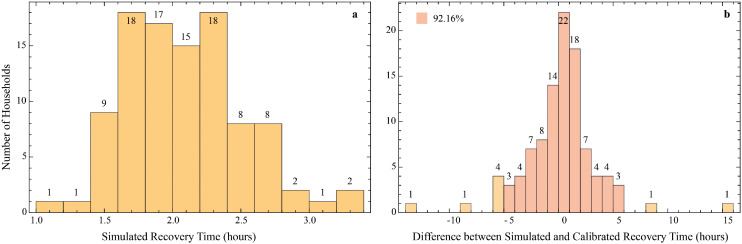
Calibration of household loss coefficient and recovery time alignment.

The average error, computed as the mean of absolute differences between simulated and empirical household recovery durations, remains within approximately two to three hours (Fig. 13a). Fig. 13b also shows the distribution of differences between simulated household recovery times and “caliloss” for one run, with 92.16 % (red area) of households within five hours. This demonstrates the model's ability to replicate observed patterns in recovery behavior.

On the other hand, we presented the model to local stakeholders, mainly local officials, and also showcased it in multiple relevant workshops. We engaged in discussions with experts from related fields and adjusted the model mechanisms based on the feedback and recommendations from these stakeholders and experts. Specific adjustments based on stakeholder input included refining the timing and targeting of emergency relief mechanisms, as well as incorporating locally relevant social network structures and informal communication patterns into the agent interaction rules. This approach is common in the verification and calibration of ABMs [60,70], and reflects broader calls for context-sensitive and participatory validation in environmental modeling [24,26]. Together, these procedures establish an empirically consistent and contextually credible foundation for assessing the system’s emergent resilience dynamics.

### Minimum number of simulations

Due to the inherent uncertainty in agent-based modeling, we calculated the minimum number of simulation runs required [71]. We examined the coefficient of variation (CV), defined as the ratio of the standard deviation to the mean, for three key outputs: global robustness, global adaptability, and recovery duration, based on 100 independent simulation runs. These CV values were calculated at the final time step of each simulation.

**Fig. 14:**
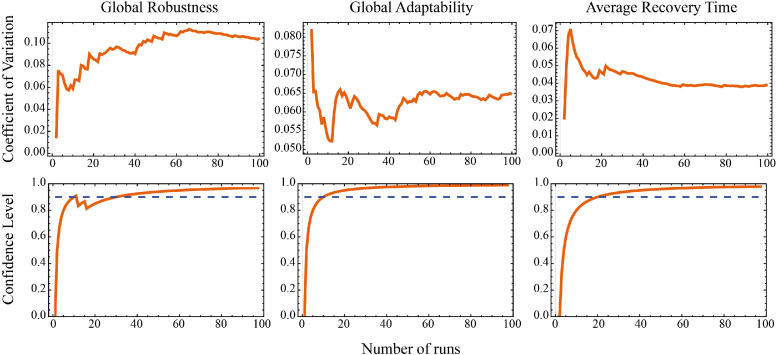
Change in coefficient of variation (CV) for key outputs (robustness, adaptability, and recovery time) with increasing number of simulation runs. Dashed lines indicate thresholds for 90 % convergence probability.

We analyzed the fluctuation range of the CV, which decreased as the number of runs, or sample size, increased, indicating increasing result stability [71]. To define a convergence threshold, we computed the probability that the difference between CVs of adjacent sample sizes falls within ±10 % of the total CV range. When the probability that the difference between adjacent CVs falls within 10 % of the CV range exceeds 90 %, the required minimum number of runs is 30 (Fig. 14). This criterion corresponds to a 90 % confidence level for the sample variation stability interval between −0.1 and +0.1.

If the probability is raised to 95 %, the minimum number of runs increases to 60, which more than doubles the computational demand. Considering computational resources and accuracy requirements, we ultimately chose 30 runs as the minimum for each experiment. Each batch of 30 simulations takes approximately 12 h to complete on the computing platform used.

### Sensitivity analysis

Similar to many agent-based models, this model employs a modified version of the one at a time (OAT) sensitivity analysis method, which is an economical and efficient approach [64,70,71]. We kept all other parameters fixed at their recommended (standard) values while varying one parameter at a time to both a higher and a lower value within a predefined range. For each parameter variation, we conducted 30 independent simulation runs. By comparing the distribution of key outputs (specifically, global robustness, global adaptability, and recovery duration) under different parameter configurations, we assessed both the direction of change (i.e., whether outputs increase or decrease) and the spread of outcomes (i.e., whether results become more stable or more dispersed), as this reflects the degree of result stability under different parameter values. This approach enables us to evaluate not only the magnitude of influence each parameter has on the system but also its impact on output variability and stability, providing insight into the model's overall robustness and sensitivity structure.

We performed sensitivity analyses on five groups of parameters, covering network structure, behavioral frequency, and policy intensity. Specifically, the first and second groups each contain one parameter related to the generation of small world networks. Since the model involves extensive network-based calculations, it is necessary to assess the sensitivity of results to network structure. The third group relates to the frequency of social network activities, while the fourth and fifth groups represent the intensities of assistance and emergency relief respectively.

Parameter settings are shown in Table 12. Behavioral frequencies are given in days. For example, a daily frequency corresponds to 100 %, every two days to 50 %, every ten days to 10 %, and so on. Additionally, to ensure the model’s computational modules operate comprehensively and fully reflect sensitivity, the value-based education module and the targeted allocation mechanisms for assistance and emergency relief recipients are enabled in all simulations.

**Table 12:** Parameter settings for sensitivity analysis experiments.

Group	Parameter	Recommended value (standard)	More complex (more random, larger networks, stronger policies, F)	No. Name	Median (M)	No. Name	Simpler (more regular, smaller network, weaker policy, S)	No. Name
1	Rewiring-probability	0.1	0.3	F1	0.2	M1	0.1	S1
2	Initial-avg-degree	4	6	F2	4	M2	2	S2
3	Frequency-learn	1	1	F3	2	M3	3	S3
	Frequency-education	14	5		10		15	
	Edu-duration	2	5		3		1	
4	Frequency-assis-local	14	10	F4	15	M4	20	S4
	Assis-local-proportion	0.3	0.5		0.3		0.1	
	Frequency-assis-hl	28	10		15		20	
	Assis-h-l-proportion	0.1	0.5		0.3		0.1	
5	Human-no-emergency relief	3	5	F5	3	M5	1	S5
	Frequency-emergency relief-local	3	1		3		5	
	Emergency relief-local-proportion	0.3	0.9		0.5		0.1	
	Frequency-emergency relief-hl	3	1		3		5	
	Emergency relief-h-l-proportion	0.1	0.9		0.5		0.1	

**Fig. 15:**
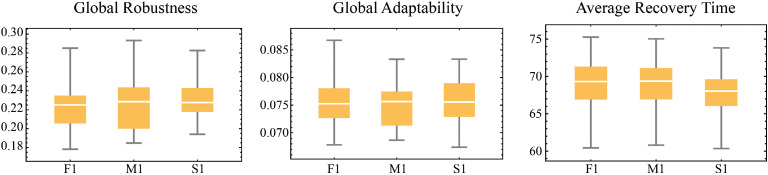
Randomness of network generation.

**Fig. 16:**
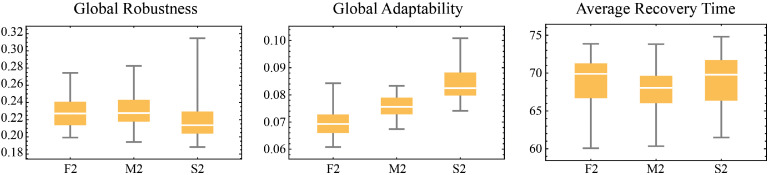
Density of network generation.

**Fig. 17:**
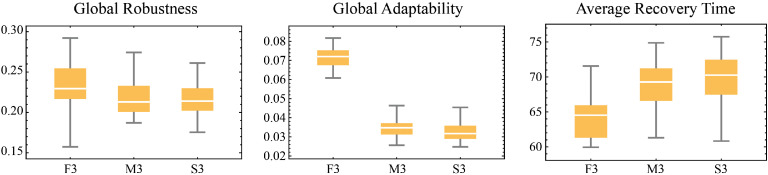
Intensity of knowledge dissemination.

**Fig. 18:**
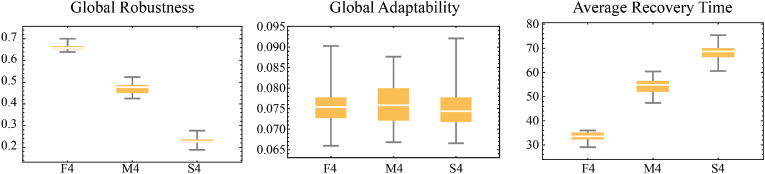
Intensity of assistance.

**Fig. 19:**
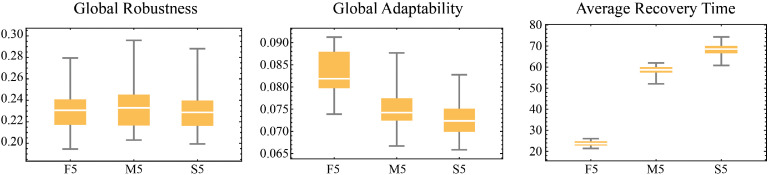
Intensity of emergency relief.

Fig. 15, Fig. 16, Fig. 17, Fig. 18, Fig. 19 present the results of these sensitivity experiments. Each figure is based on the final outputs aggregated across 30 independent simulation runs per parameter setting. Box plots are used to show the distribution of the outcomes across runs, including the median (central line), interquartile range (box), and overall data spread (whiskers), with outliers indicated when present. This visualization facilitates comparative analysis of output variability, identifies potential outliers, and reveals central tendencies across scenarios, thereby supporting the interpretation of parameter influence on model behavior [71,72].

In the first group, we tested the parameter “Rewiring-probability”, which represents the randomness of network generation. In each panel of Fig. 15, the left side corresponds to more random networks (F1), while the right side represents more regular networks (S1). The analysis of three key outputs is as follows. First, global robustness shows a slight increase as network randomness increases. Second, global adaptability is not significantly affected by network randomness, with relatively consistent distributions across groups. Third, the average recovery time shows a decreasing trend as randomness increases. Overall, the degree of network randomness does not appear to be a determining factor in shaping the three key model outputs.

In the second group, we tested the parameter “Initial-avg-degree”, which represents the density of the network. In each panel of Fig. 16, the left side indicates denser networks (F2), while the right side corresponds to sparser networks (S2). The analysis of three key outputs is summarized as follows. First, global robustness shows a wider possible range and a general decreasing trend as the network becomes sparser. Second, global adaptability is higher under sparse network conditions. This may be because connections in sparse networks are more selective, leading to more efficient information transmission and avoiding the redundancy or competition that can occur in dense networks. Third, the average recovery time remains relatively stable. The second group of results suggests that sparse networks can more effectively enhance system adaptability but may introduce greater instability in robustness, potentially leading to lower overall robustness. Therefore, in such cases, increased adaptability does not necessarily lead to reduced losses.

In the third group, we tested parameters related to the intensity of knowledge dissemination, including “learning frequency”, “education frequency”, and “duration of education”, in order to explore how knowledge dissemination affects model outputs. In each panel of Fig. 17, a position farther to the left indicates higher knowledge dissemination intensity. The analysis of three key outputs is as follows. First, global robustness tends to decrease and becomes more concentrated as the intensity of knowledge dissemination declines. This suggests that knowledge dissemination introduces more uncertainty into the development of robustness among individuals in the model. Second, an increase in global adaptability is mainly observed when learning frequency and education frequency are relatively high, indicating that stronger knowledge dissemination can promote adaptability under certain conditions. Third, average recovery time increases with lower knowledge dissemination intensity. These results indicate that while enhancing knowledge dissemination can improve adaptability, the effect remains relatively limited, and it also introduces greater uncertainty in the development of robustness. Overall, stronger knowledge dissemination can help reduce losses to some extent [73,74].

The fourth group examined the intensity of government assistance, including the frequency of assistance and the proportion of households receiving it. In each panel of Fig. 18, the levels from left to right represent high, medium, and low assistance intensity. The analysis of three key outputs is summarized as follows. First, government assistance intensity has a significant impact on global robustness. Higher levels of assistance result in higher global robustness. Second, the impact on global adaptability is less pronounced, although there is a slight decreasing trend as assistance increases. This is because, in the model, government assistance is also constrained by the internal resource status of the community, which depends on contributions made by individuals through voluntary actions. Third, assistance intensity has a substantial effect on average recovery time. These results are consistent with real-world observations and empirical studies, confirming that the strength of government assistance plays a vital role in enhancing system resilience and improving recovery efficiency [75].

The fifth group focused on the intensity of government emergency relief efforts, including the frequency of emergency relief actions, the proportion of households receiving emergency relief, and the average manpower input. In each panel of Fig. 19, the levels from left to right represent high, medium, and low emergency relief intensity. The analysis of three key outputs is summarized as follows. First, the impact of emergency relief intensity on global robustness is limited, with only minor variations in the global robustness indicator across high, medium, and low levels. Second, emergency relief intensity has a significant effect on global adaptability, which increases as emergency relief efforts become stronger. Third, a negative correlation is observed with average recovery time, where higher emergency relief intensity leads to shorter recovery periods, but the effect weakens with lower intensity. Emergency relief intensity has a significant influence on global adaptability and recovery time, and the decline in adaptability is closely associated with increased losses [76,77]. However, its impact on global robustness remains limited.

These sensitivity analyses provide a robust assessment of the model’s structural stability under varying assumptions and confirm that certain policy parameters, such as emergency relief intensity, have stronger and more consistent effects on resilience outcomes. In particular, higher levels of government assistance reduce average recovery time by around 50 % and increase global robustness by about 30 %, while stronger emergency relief measures primarily enhance adaptability (approximately 10 %) and shorten recovery time, with only limited influence on robustness. By contrast, network structure and knowledge dissemination exert more nuanced effects: sparser networks increase adaptability but reduce robustness stability, and higher knowledge dissemination approximately doubles adaptability but introduces greater variability in robustness. Together, these findings demonstrate both the relative weight of different policy levers and the trade-offs inherent in shaping community flood resilience.

## Limitations

While the current model provides valuable insights into community flood resilience, it also has several limitations that suggest directions for future improvement. Future research could expand and refine the model in several important directions. First, although the model is empirically grounded, the household survey data used for parameterization are limited in spatial and temporal coverage, which may constrain the generalizability of the findings. Expanding the empirical basis with cross-regional or longitudinal data would strengthen the robustness of the model. Second, the integration of hydrological, governance, and behavioral processes inevitably involves abstraction and simplification. Certain complex governance interactions and social learning mechanisms are not fully captured, highlighting opportunities for future extensions to better represent these dynamics. Third, incorporating the decision-making processes of multi-level governments would allow for a more realistic representation of interactions and coordination across different tiers of governance. Finally, the model could be extended to include repeated shocks, compound disasters, and the longer-term dynamics of adaptive evolution, thereby enhancing its ability to capture resilience over time.

## Ethics statements

The household interview data used in the study were obtained with the consent of the respondents. Ethical approval was granted by the Ethics Committee of LMU Munich (Project No 22-0450).

## Supplementary material *and/or* additional information [OPTIONAL]

The model code, relevant files, and necessary data can be accessed from: https://www.comses.net/codebase-release/db311523-94f9-4044-8dcc-c208f7707486/

## CRediT authorship contribution statement

**Wenhan Feng:** Conceptualization, Methodology, Software, Writing – original draft, Visualization, Investigation, Validation. **Liang Emlyn Yang:** Supervision, Writing – review & editing. **Mei Ai:** Conceptualization, Methodology, Software, Writing – original draft, Visualization, Investigation, Validation. **Siying Chen:** Conceptualization, Methodology, Software, Writing – original draft, Visualization, Investigation, Validation. **Ziyao Wang:** Data curation. **Wenhao Wu:** Conceptualization, Methodology, Software, Writing – original draft, Visualization, Investigation, Validation. **Junxu Chen:** Supervision, Writing – review & editing. **Yiping Fang:** Supervision, Writing – review & editing. **Yun Xu:** Supervision, Writing – review & editing. **Matthias Garschagen:** Supervision, Writing – review & editing.

## Declaration of interests

The authors declare that they have no known competing financial interests or personal relationships that could have appeared to influence the work reported in this paper.

## Data Availability

I have schared the link of my data and code at additional information
